# High‐resolution analysis of individual spike peptide‐specific CD4 ^+^ T‐cell responses in vaccine recipients and COVID‐19 patients

**DOI:** 10.1002/cti2.1410

**Published:** 2022-08-09

**Authors:** Hendrik Karsten, Leon Cords, Tim Westphal, Maximilian Knapp, Thomas Theo Brehm, Lennart Hermanussen, Till Frederik Omansen, Stefan Schmiedel, Robin Woost, Vanessa Ditt, Sven Peine, Marc Lütgehetmann, Samuel Huber, Christin Ackermann, Melanie Wittner, Marylyn Martina Addo, Alessandro Sette, John Sidney, Julian Schulze zur Wiesch

**Affiliations:** ^1^ Infectious Diseases Unit, 1. Department of Medicine University Medical Center Hamburg‐Eppendorf Hamburg Germany; ^2^ German Center for Infection Research (DZIF) Partner Site Hamburg‐Lübeck‐Borstel‐Riems Hamburg Germany; ^3^ Department of Tropical Medicine Bernhard Nocht Institute for Tropical Medicine Hamburg Germany; ^4^ Institute of Transfusion Medicine University Medical Center Hamburg‐Eppendorf Hamburg Germany; ^5^ Institute of Medical Microbiology, Virology and Hygiene University Medical Center Hamburg‐Eppendorf Hamburg Germany; ^6^ Center for Infectious Disease and Vaccine Research La Jolla Institute for Immunology (LJI) La Jolla CA USA

**Keywords:** B.1.1.529, CD4^+^ T cells, MHC class II, SARS‐CoV‐2, spike protein, vaccines

## Abstract

**Objectives:**

Potential differences in the breadth, distribution and magnitude of CD4^+^ T‐cell responses directed against the SARS‐CoV‐2 spike glycoprotein between vaccinees, COVID‐19 patients and subjects who experienced both ways of immunisation have not been comprehensively compared on a peptide level.

**Methods:**

Following virus‐specific *in vitro* cultivation, we determined the T‐cell responses directed against 253 individual overlapping 15‐mer peptides covering the entire SARS‐CoV‐2 spike glycoprotein using IFN‐γ ELISpot and intracellular cytokine staining. *In vitro* HLA binding was determined for selected peptides.

**Results:**

We mapped 955 single peptide‐specific CD4^+^ T‐cell responses in a cohort of COVID‐19 patients (*n* = 8), uninfected vaccinees (*n* = 16) and individuals who experienced both infection and vaccination (*n* = 11). Patients and vaccinees (two‐time and three‐time vaccinees alike) had a comparable number of CD4^+^ T‐cell responses (median 26 vs. 29, *P* = 0.7289). Most of these specificities were conserved in B.1.1.529 and the BA.4 and BA.5 sublineages. The highest magnitude of these *in vitro* IFN‐γ CD4^+^ T‐cell responses was observed in COVID‐19 patients (median 0.35%), and three‐time vaccinees showed a higher magnitude than two‐time vaccinees (median 0.091% vs. 0.175%, *P* < 0.0001). Twelve peptide specificities were each detected in at least 40% of subjects. *In vitro* HLA binding showed promiscuous presentation by DRB1 molecules for several peptides.

**Conclusion:**

Both SARS‐CoV‐2 infection and vaccination prime broadly directed T‐cell responses directed against the SARS‐CoV‐2 spike glycoprotein. This comprehensive high‐resolution analysis of spike peptide specificities will be a useful resource for further investigation of spike‐specific T‐cell responses.

## Graphical abstract

Individual CD4^+^ and CD8^+^ T‐cell responses directed against a total of 253 overlapping 15‐mer spike‐specific peptides were mapped in a cohort of COVID‐19 patients (*n* = 8), uninfected vaccinees (*n* = 16) and individuals who experienced both infection and vaccination (*n* = 11). We found that patients and vaccinees (two‐time and three‐time vaccinees alike) had a comparable number of CD4^+^ T‐cell responses (median 26 vs. 29, *P* = 0.7289) but differential magnitudes of these *in vitro* responses (median 0.35% vs. 0.12% IFN‐γ^+^ of CD4^+^ T cells, *P* < 0.0001). Most of the recognized specificities were conserved in the B.1.1.529 (omicron) Variant of Concern (VoC), and its sublineages and several peptides showed promiscuous presentation by DRB1 molecules in *in vitro* HLA‐binding assays.

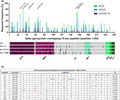

## Introduction

The severe acute respiratory syndrome coronavirus type 2 (SARS‐CoV‐2) is the third coronavirus in recent years causing symptoms more severe than the common cold. In severe cases, coronavirus disease 2019 (COVID‐19) is characterised by immunologic dysregulation and hyperinflammation.^1^, ^2^, ^3^, ^4^ Lethal courses are observed in elderly patients and those with comorbidities, most notably diabetes, hypertension and obesity.^4^, ^5^, ^6^ Vaccine‐induced immunity against SARS‐CoV‐2 has been shown to prevent severe and lethal courses of COVID‐19 or even infection.^7^, ^8^, ^9^

Most vaccination strategies against SARS‐CoV‐2 target the spike glycoprotein as one of the main viral immunogenic structures. The spike glycoprotein is a structural protein of SARS‐CoV‐2 and is located at the surface of the virus.^10^ It consists of 1273 amino acids and has several distinct domains.^11^ Functionally, the spike glycoprotein forms part of the viral envelope and mediates the binding of the virus particle to the host cell via the interaction of the receptor‐binding domain (RBD) with the angiotensin‐converting enzyme 2 (ACE2).^12^, ^13^ The fusion peptide (FP) of the spike glycoprotein mediates the entry into the host cell by disrupting the phospholipid bilayer.^14^

SARS‐CoV‐2 vaccines aim to induce a robust neutralising antibody response, and a specific T‐cell memory to establish protective immunity.^15^ While the T‐cell response has been shown to modulate disease severity and clinical outcome,^16^, ^17^, ^18^, ^19^ it has been proposed that a neutralising antibody response against the spike glycoprotein can prevent severe symptoms or even infection.^20^, ^21^, ^22^ Previous findings indicate that the quality of the antibody response is dependent on the vaccination regimen^23^, ^24^, ^25^ and the application of booster vaccinations.^26^, ^27^, ^28^, ^29^

Great efforts have been made to study spike‐specific T cells, but the knowledge about the exact number and location of individual specificities of infection‐ and vaccine‐induced T‐cell responses is still limited and needs to be increased. To date, there have been bioinformatics and *in silico* approaches to identify immunodominant SARS‐CoV‐2 epitopes.^30^, ^31^, ^32^ Consequently, research has mainly focused on predicted epitopes or used pooled peptides to stimulate T cells. These approaches are limited by the relatively low resolution resulting from the approach of investigating peptide pools of proteins, subunits or domains.

Until now, there has not been a systematic investigation of the T‐cell responses directed against the SARS‐CoV‐2 spike glycoprotein on a single peptide level comparing vaccinees versus COVID‐19 patients. Using 253 overlapping 15‐mer peptides covering the whole spike glycoprotein and a very sensitive *in vitro* approach, we determined the breadth, magnitude and specificity of dominant SARS‐CoV‐2 spike glycoprotein‐specific T‐cell responses after SARS‐CoV‐2 infection, vaccination or a combination of both. Our results provide evidence for the efficacy of vaccines to induce strong, long‐lasting and possibly cross‐SARS‐CoV‐2‐variant specific T‐cell responses and could be used to optimise future vaccines. Furthermore, high‐resolution data about the localisation of indivudal epitopes within the proteins of SARS‐CoV‐2 are important to evaluate the potential influence of viral mutations in immunodominant regions on anti‐SARS‐CoV‐2 immunity. This large and unprecedented, high‐resolution data set on the spike‐specific T‐cell response will facilitate future investigations on COVID‐19 pathogenesis, and natural and vaccine‐induced T‐cell immunity against SARS‐CoV‐2. It provides additional information on novel spike peptide specificities for the development of peptide–MHC class II multimers.

## Results

### Patient characteristics

Enrolment of study participants was carried out at the University Medical Center Hamburg‐Eppendorf between May 2021 and February 2022; the clinical characteristics are summarised in Table 1. The subjects were stratified according to their infection and vaccination status. Infection was confirmed by current or prior detection of SARS‐CoV‐2 by polymerase chain reaction (PCR) from oropharyngeal and/or nasopharyngeal swabs.^33^ Disease severity was graded according to the WHO progression scale.^34^ Infection was ruled out if the subjects never tested positive for SARS‐CoV‐2 by PCR testing and had a negative SARS‐CoV‐2 nucleocapsid protein antibody titre.^35^ The time since the last immunising event was defined for each patient as the time passed since the last SARS‐CoV‐2 detection by PCR or the last vaccination administered. Further details on single patient characteristics, such as infection with a particular SARS‐CoV‐2 viral variant, can be found in Supplementary table 1.

**Table 1:** Clinical characteristics of the study cohort. Data are expressed as absolute numbers or mean with either range or percentage. Disease severity was classified according to the WHO Progression Scale. Immunising events were defined as the diagnosis of SARS‐CoV‐2 infection by PCR from a nasopharyngeal swab or administration of a vaccine

	SARS‐CoV‐2 infection	SARS‐CoV‐2 vaccination	SARS‐CoV‐2 infection and vaccination
	(COVID‐19)	(V2/V3)	(IV/VI)
	*n* = 8	*n* = 16	*n* = 11
Age in years (range)	54.9 (21–82)	35.3 (21–56)	54 (23–95)
Sex at birth			
Male (%)	7 (87.5%)	6 (37.5%)	7 (63.64%)
Female (%)	1 (12.5%)	10 (62.5%)	4 (36.36%)
Disease severity			
Uninfected – WHO 0 (%)	–	16 (100%)	–
Ambulatory mild disease – WHO 1–3 (%)	1 (12.5%)	–	6 (54.55%)
Hospitalised: moderate disease – WHO 4–5 (%)	2 (25%)	–	4 (36.36%)
Hospitalised: severe disease – WHO 6–9 (%)	4 (50%)	–	–
Unknown	1 (12.5%)	–	1 (9.09%)
Vaccination regime			
1 dose mRNA (%)	–	–	2 (18.18%)
1 dose vector (%)	–	–	2 (18.18%)
2 doses mRNA/mRNA (%)	–	4 (25%)	3 (27.27%)
2 doses vector/mRNA (%)	–	5 (31.25%)	1 (9.1%)
3 doses mRNA/mRNA/mRNA (%)	–	4 (25%)	–
3 doses vector/mRNA/mRNA (%)	–	2 (12.5%)	–
4 doses vector/mRNA/mRNA/mRNA (%)	–	1 (6.25%)	–
Unknown	–	–	3 (27.27%)
Time since last immunising event in days (range)	88.0 (8–448)	60.7 (3–196)	80.1 (1–179)

In total, 35 participants with a median age of 45 years (range 21–95 years) were recruited at a median time of 40 days (range 1–448 days) after the last immunising event. 43% of the subjects identified as female. We included eight unvaccinated individuals with acute or resolved COVID‐19. Of the 11 individuals, who reported both infection and vaccination, six received at least one dose of a vaccine after their convalescence (‘IV’), and five contracted COVID‐19 after their vaccination (‘VI’). Of the 16 uninfected individuals, nine reported two (‘V2’) and seven reported three or four vaccination doses (‘V3’). The applied vaccines were either based on mRNA technology (BNT‐162b2 or mRNA‐1273) or were vector‐based (ChAdOx1 nCoV‐19). The heterogenicity of applied vaccination regimens and the number of doses were a result of the repeated adjustments of the vaccination guidelines in Germany, which recommended a booster vaccination for certain individuals from October 2021 onwards.^36^

Molecular HLA typing of the DRB1 locus was available for 30 of the 35 study participants. We compared the pattern of the HLA‐DRB1 alleles of our study cohort with representative population data from the German Bone Marrow Donor File (DKMS) acquired from the allele frequency net database (AFND, http://www.allelefrequencies.net).^37^ As shown in Supplementary figure 1, many DRB1 alleles of our study population matched the representative population data. DRB molecules that were relatively underrepresented in our study with a difference of ≥ 1.5% in allele frequencies compared with the DKMS data were DRB1*03:01, DRB1*11:01 and DRB1*13:02. However, the differences in allele frequency did not exceed 4.3%, indicating a good representation of the HLA distribution in western Europe in our study cohort.

### Comparable breadth but a higher magnitude of the spike‐specific CD4 ^+^ T‐cell response in infected individuals

In this study, we aimed to assess the breadth of the T‐cell response and its specificities within the SARS‐CoV‐2 spike glycoprotein. We investigated the T‐cell response after *in vitro* spike peptide‐specific cell culture to single 15‐mer peptides of the SARS‐CoV‐2 spike glycoprotein using IFN‐γ ELISpot, as has been done before for the sensitive and high‐resolution characterisation of viral T‐cell epitopes.^35^, ^38^, ^39^, ^40^, ^41^, ^42^ Each positive ELISpot response was confirmed and classified as a CD4^+^ or CD8^+^ T‐cell response by intracellular cytokine staining (ICS) for IFN‐γ after restimulation with the respective single peptide.

**Figure 1:**
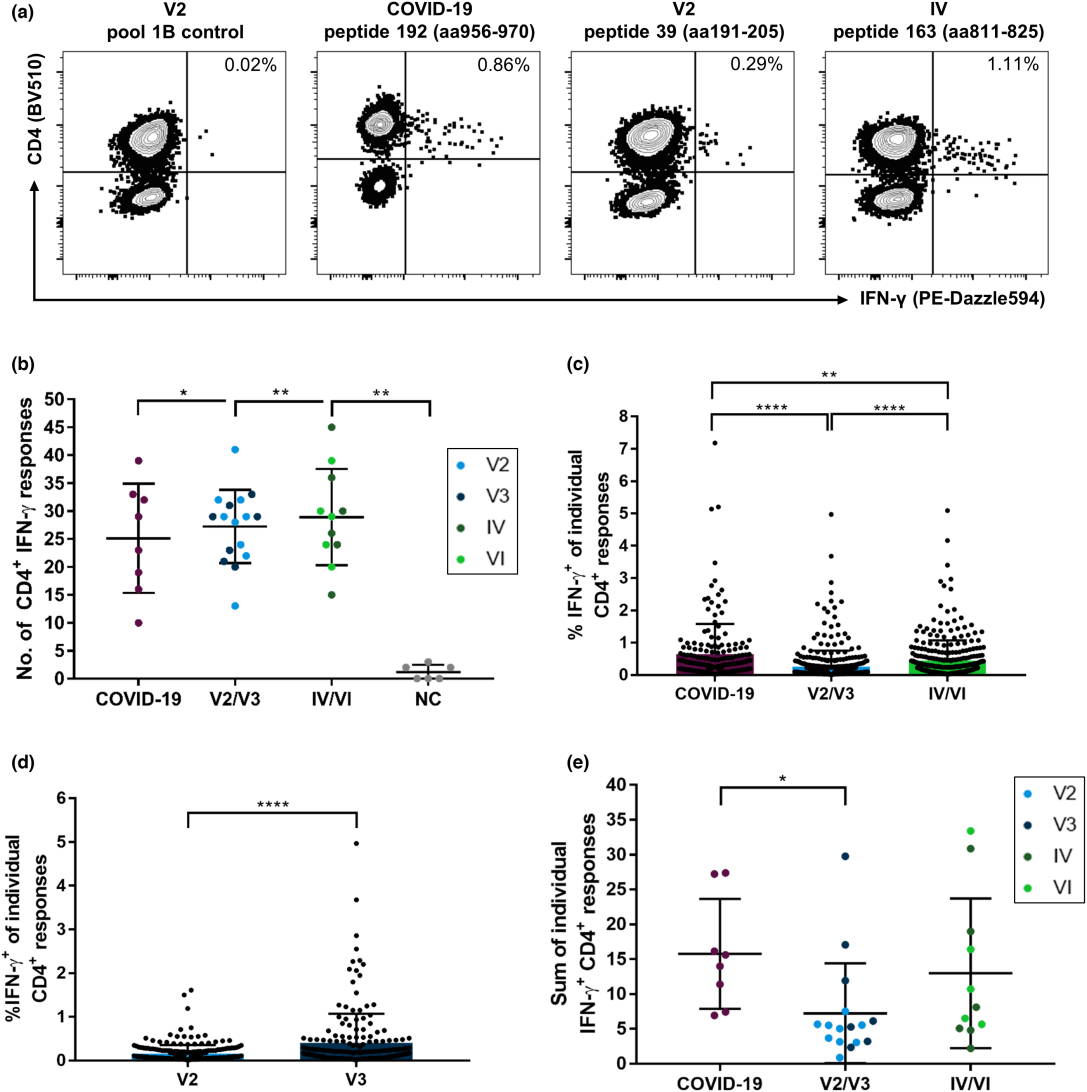
Number and magnitude of IFN‐γ CD4^+^ T‐cell responses directed against individual SARS‐CoV‐2 spike glycoprotein peptides of a comprehensive peptide set detected in COVID‐19 patients, COVID‐19‐naive individuals after vaccination or individuals with a combination of both. PBMCs of the participants were expanded *in vitro* with pools of overlapping spike peptides in the presence of anti‐CD28/anti‐CD49d antibodies and IL‐2 for 14 days before restimulation with single peptides. Cells were analysed with single‐peptide IFN‐γ ELISpot assays and validated with intracellular cytokine staining for IFN‐γ. IFN‐γ T‐cell responses mainly composed of CD4^+^ T‐cell responses in all groups **(a)**. While the participants in all study groups recognised more specificities than pre‐pandemic, healthy individuals (NC), there were no significant differences in the number **(b)** but in the magnitude of CD4^+^ T‐cell responses **(c)** between the study groups. Within the vaccination group, spike peptides elicited stronger IFN‐γ T‐cell responses in individuals who received a booster vaccination **(d)**. The summation of IFN‐γ T‐cell responses showed a higher IFN‐γ production in individuals with COVID‐19 than in vaccinated individuals **(e)**. Data are expressed as mean with standard deviation. *P < 0.05; **P < 0.01; ***P < 0.001; and ****P < 0.0001.

In Figure 1a, representative flow cytometric plots for IFN‐γ^+^ spike‐specific CD4^+^ T‐cell responses are shown. We could only detect very few spike‐specific CD8^+^ T‐cell responses in most individuals (Supplementary figure 2). Most of the elicited IFN‐γ responses were CD4^+^ T‐cell responses in the flow cytometric analyses. We observed a total of 955 individual CD4^+^ and 220 individual CD8^+^ T‐cell responses in the 35 participants. While we could not detect spike‐specific CD8^+^ T‐cell responses in every participant (median 6; range 0–15), CD4^+^ T cells of each individual recognised at least 10 different peptide specificities (median 29; range 10–45).

We detected a similar breadth of the CD4^+^ T‐cell response in terms of the number of peptides recognised in unvaccinated COVID‐19 patients, vaccinated individuals (‘V2/V3’) and patients with a combination of vaccination and infection (‘IV/VI’) (Figure 1b). While individuals with acute or recovered COVID‐19 showed a median of 26 different spike peptide‐specific CD4^+^ T‐cell responses (range 10–39), vaccinated individuals responded to a median of 29 CD4^+^ T‐cell specificities (range 13–41). IV/VI individuals showed a median of 29 responses (range 15–45; IV/VI vs. COVID‐19, *P* = 0.4788; IV/VI vs. V2/V3, *P* = 0.6878). Notably, we could only detect very few CD4^+^ T‐cell responses (median 1; range 0–3) in samples of six pre‐pandemic, healthy individuals (‘NC’). Interestingly, for all study participants, the number of CD4^+^ T‐cell responses correlated with the number of CD8^+^ T‐cell responses (*r* = 0.3558, *P* = 0.0359) (Supplementary figure 3a). Further stratification of the groups by different vaccination protocols (homologous mRNA/mRNA vaccination vs. heterologous vector/mRNA vaccination) or clinical features (immunosuppression and disease severity graded by the WHO progression scale) did not reveal any notable differences. However, smaller potential differences might have been hidden by the pooling of individuals and need to be prospectively analysed using larger cohorts (Supplementary figure 3b–e).

The magnitude, defined as the proportion of IFN‐γ^+^ out of all CD4^+^ T cells in response to the peptides, markedly differed between the study groups (Figure 1c). For the unvaccinated COVID‐19 patients, we detected a median of 0.35% (range 0.024–7.18%) IFN‐γ^+^ cells per CD4^+^ T‐cell response. Therefore, the magnitude in the COVID‐19 group was significantly elevated compared with that in the V2/V3 group and the IV/VI group, which showed a median of 0.12% (range 0.02–4.97%) and 0.24% (range 0.02–4.17%) IFN‐γ^+^ cells per peptide, respectively (COVID‐19 vs. V2/V3, *P* < 0.0001; COVID‐19 vs. IV/VI, *P* < 0.0001; and V2/V3 vs. IV/VI, *P* = 0.0031). Of note, within the vaccination group, the individuals with only two vaccinations showed a significantly lower magnitude (median 0.091%, range 0.022–1.61%, *P* < 0.0001) of the spike‐specific CD4^+^ T‐cell response measured by IFN‐γ^+^ after restimulation in the ICS than the individuals who had already received a third vaccination (median 0.175%, range 0.02–4.97%) (Figure 1d). This was not associated with the time after the last vaccination (V2 median 49 days, range 26–196 days; V3 median 28 days, range 3–52 days, *P* > 0.05). Additionally, we observed a correlation (*r* = 0.525, *P* = 0.0024) between the mean magnitude of CD4^+^ and CD8^+^ T‐cell responses (Supplementary figure 3f).

When we summed up all single‐peptide spike‐specific CD4^+^ T‐cell responses measured for each individual (Figure 1e), the proportion of IFN‐γ^+^ CD4^+^ T cells of each subject tended to be higher in the COVID‐19 (median 14.796) and the IV/VI (median 8.117) groups than in the V2/V3 (median 5.385) group. This only reached statistical significance for the comparison of the COVID‐19 and the V2/V3 groups (*P* = 0.0045; V2/V3 vs. IV/VI, *P* = 0.0797; COVID‐19 vs. IV/VI, *P* = 0.3511).

Individual spike‐specific CD4^+^ T‐cell responses seemed to be long‐lasting since we could still detect dominant responses 196 days (individual HH‐SP‐35) after the last vaccination and 448 days (individual HH‐SP‐08) after resolved infection. We performed a correlation analysis between the number of recognised epitopes and the average magnitude of the CD4^+^ and CD8^+^ IFN‐γ^+^ response with the anti‐spike antibody titres, with the time since the last immunising event and with age (Supplementary figure 4a–c). The analyses did not reveal any statistically significant associations, except for a correlation between age and the average magnitude of spike‐specific CD4^+^ responses (*r* = 0.4128, *P* = 0.0014). Moreover, there were no significant differences with regard to the number of responses or the magnitude of the response between male and female individuals (Supplementary figure 5).

B.1.1.529 and all previously circulating Variants of Concern (VoC)s and its lineages under monitoring (LUM) show several mutations in the spike glycoprotein.^43^, ^44^ This raises the question of whether vaccination with wild‐type spike glycoprotein primes sufficient cross‐reactive T‐cell responses.^27^, ^45^ Incidentally, subject HH‐SP‐26, who was infected with the B.1.1.529.1 variant, showed a similar number of spike‐specific CD4^+^ T‐cell responses compared to patients infected with other virus variants. Even under the assumption that there was no cross‐reactivity between a wild‐type peptide specificity that differs at any amino acid position for the B.1.1.529 VoC, there would be considerable T‐cell responses: Even if all peptides with any mutational change were not counted as a potential response, there would still be at least seven responses attributable in conserved regions and peptide sequences for every patient (median 21; range 7–35) (Supplementary figure 6a, b). A similar number of responses were conserved in all of the VoC–LUMs (Supplementary figure 6b). The CD8^+^ T‐cell responses showed similar results with a median of 4 responses per patient after the subtraction of B.1.1.529 VoC‐mutated peptide sequences (*P* < 0.0001; range 0–13; data not shown) and similar results for its LUMs.

Taken together, our results show that vaccinated and infected individuals alike broadly recognise specificities within the SARS‐CoV‐2 spike glycoprotein with every participant having at least 10 CD4^+^ T‐cell responses. While the number of recognised epitopes was similar regardless of clinical features, the frequency of IFN‐γ‐producing CD4^+^ T cells was significantly increased in infected individuals compared with that in vaccinated individuals after *in vitro* cultivation. Booster vaccination tended to lead to higher magnitudes than two‐times vaccination.

### Epitope distribution within the SARS‐CoV‐2 spike glycoprotein

**Figure 2:**
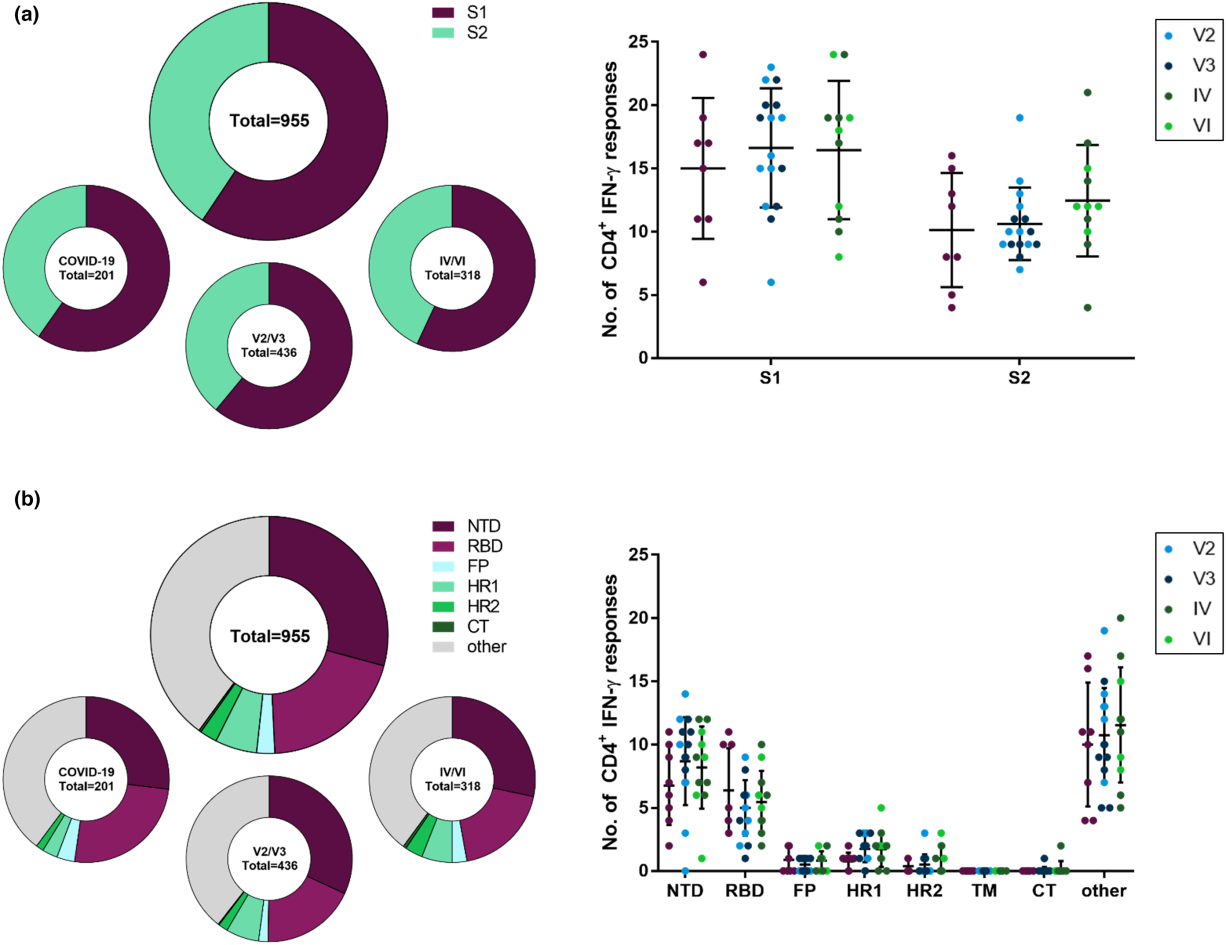
Distribution of peptide‐specific CD4^+^ IFN‐γ T‐cell responses within the subunits and domains of the SARS‐CoV‐2 spike glycoprotein. Distribution of CD4^+^ IFN‐γ T‐cell responses in the S1 and S2 subunits of the spike glycoprotein **(a)** and the functional domains of the spike glycoprotein **(b)** were analysed as percentages of all responses and absolute numbers for all study participants and subdivided for the COVID‐19, V2/V3 and IV/VI groups. NTD, N‐terminal domain; RBD, receptor‐binding domain; FP, fusion peptide; HR1, heptapeptide repeat sequence 1; HR2, heptapeptide repeat sequence 2; CT, cytoplasmic tail. Data are expressed as mean with standard deviation.

Altogether, the 253 overlapping 15‐mer peptides elicited 955 CD4^+^ IFN‐γ T‐cell responses in our cohort of 35 participants, and a total of 174 of the peptide specificities (68.8%) were targeted by at least one participant. The spike‐specific T‐cell response has been subdivided in the literature into two subunits S1 (aa14–685, corresponding to peptides 1–136) and S2 (aa686–1273, corresponding to peptides 137–253). In the S1 subunit, we found 567 (59.4%), and in the S2 subunit, 388 (40.6%) individual spike‐specific CD4^+^ T‐cell responses (Figure 2a). Generally, we found significantly more responses in the S1 subunit than in the S2 subunit, not only for all study groups (COVID‐19, *P* = 0.0078; V2/V3, *P* < 0.0001; and IV/VI, *P* = 0.0352) but also for most individual participants (Supplementary figure 7a, b). This result was significant also after adjustment for the different sizes of the subunits for individuals of the COVID‐19 and V2/V3 groups but not the IV/VI group (data not shown). Following our previous observations, individuals of the V2/V3 and IV/VI groups had more responses located within the global spike glycoprotein (Figure 1b) and in both the S1 and S2 subunits than the individuals with COVID‐19 (S1: 16.6, 16.5 and 15 responses; S2: 10.6, 12.5 and 10.1 responses; Figure 2a). In the S1 subunit, 94 different peptides accounted for the responses detected, and each of these was recognised by a median of 12.86% of participants. The responses in the S2 subunit were elicited by 80 peptides with a median of 8.57% of responding patients (Supplementary figure 7c).

To further characterise the localisation of the responses within the spike glycoprotein, the S1 and S2 subunits were subdivided into the commonly described functional domains. Within the S1 subunit, the N‐terminal domain (NTD, peptides 2–60, aa14–305) and the receptor‐binding domain (RBD, peptides 63–107, aa319–541) have previously been defined. Within the S2 subunit, the fusion peptide (FP, peptides 156–161, aa788–806), the heptapeptide repeat sequences 1 (HR1, peptides 183–197, aa912–984) and 2 (HR2, peptides 233–242, aa1163–1213), the transmembrane domain (TM, peptides 243–247, aa1213–1237) and the cytoplasmic tail (CT, peptides 248–253, aa1237–1273) are known.^11^ Every patient showed spike‐specific CD4^+^ T‐cell IFN‐γ responses to peptides of at least two different functional domains. The functional domain that accounted for most responses was the NTD (283, 29.63%), followed by the RBD (191, 20%) and the HR1 (53, 5.55%). However, most responses (379, 39.69%) were located outside any of the functional domains (Figure 2b and Supplementary figure 8a). Of note, the peptides within TM did not elicit any response in any of the participants. The responses in the RBD were distributed among 29 of the 45 peptides (Supplementary figure 8b), and the CD4^+^ T cells of every patient recognised at least one peptide specificity located within the RBD (range 1–11). This was not the case for any other functional domain.

Interestingly, peptides that were more frequently recognised by the study participants' CD4^+^ T cells were more likely to be also recognised by CD8^+^ T cells (*r* = 0.7185, *P* < 0.0001). In addition, there was a statistically significant correlation between the magnitudes of the CD4^+^ and CD8^+^ T‐cell responses (*r* = 0.5651, *P* < 0.0001) (Supplementary figure 9).

These results suggest more frequent targeting of the S1 subunit and its functional domains in both SARS‐CoV‐2‐infected patients and vaccinees than that of the S2 subunit. The RBD was the most broadly recognised functional domain in our study with at least one spike peptide‐specific CD4^+^ T‐cell response in every patient and the highest recognition rates for its immunogenic peptides.

### Frequently recognised peptide specificities within the spike glycoprotein show promiscuous binding to a diverse set of HLA‐DRB1 molecules

**Figure 3:**
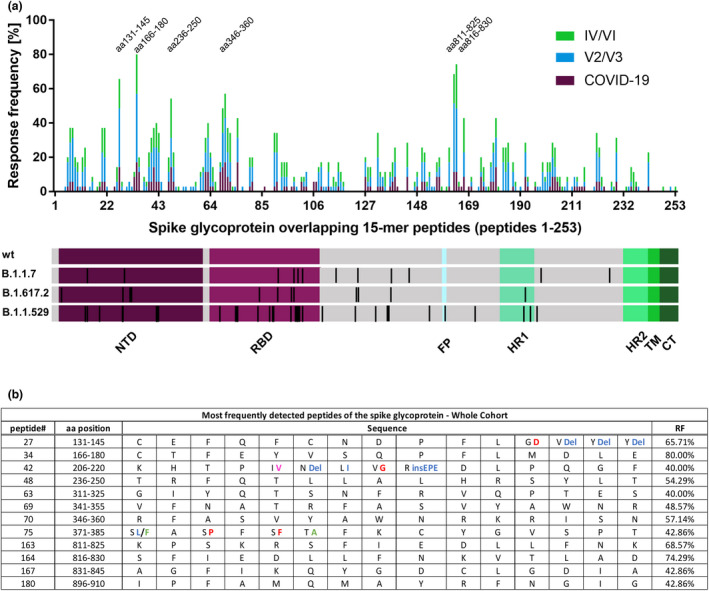
Response frequency of individual spike 15‐mer peptides and identification of the most frequently detected peptides. Individual peptide‐specific CD4^+^ T‐cell response frequencies of SARS‐CoV‐2 infected, vaccinated and individuals with both vaccination and infection to each of the 253 overlapping 15‐mer peptides covering the spike glycoprotein **(a)**. Peptide numbers, corresponding amino acid positions and sequence, and the response frequency of the most frequently detected peptides **(b)**. Mutations in the B.1.1.529 LUMs are highlighted: Mutations only found in BA.1 are depicted in blue, mutations found in BA.1, BA.2, BA.2.12, BA.4/5 and BA.2.75 are depicted in red, and mutations found in BA.2, BA.2.12, BA.4/5 and BA.2.75 are depicted in green. Mutations only found in BA.2.75 are depicted in pink.

**Figure 4:**
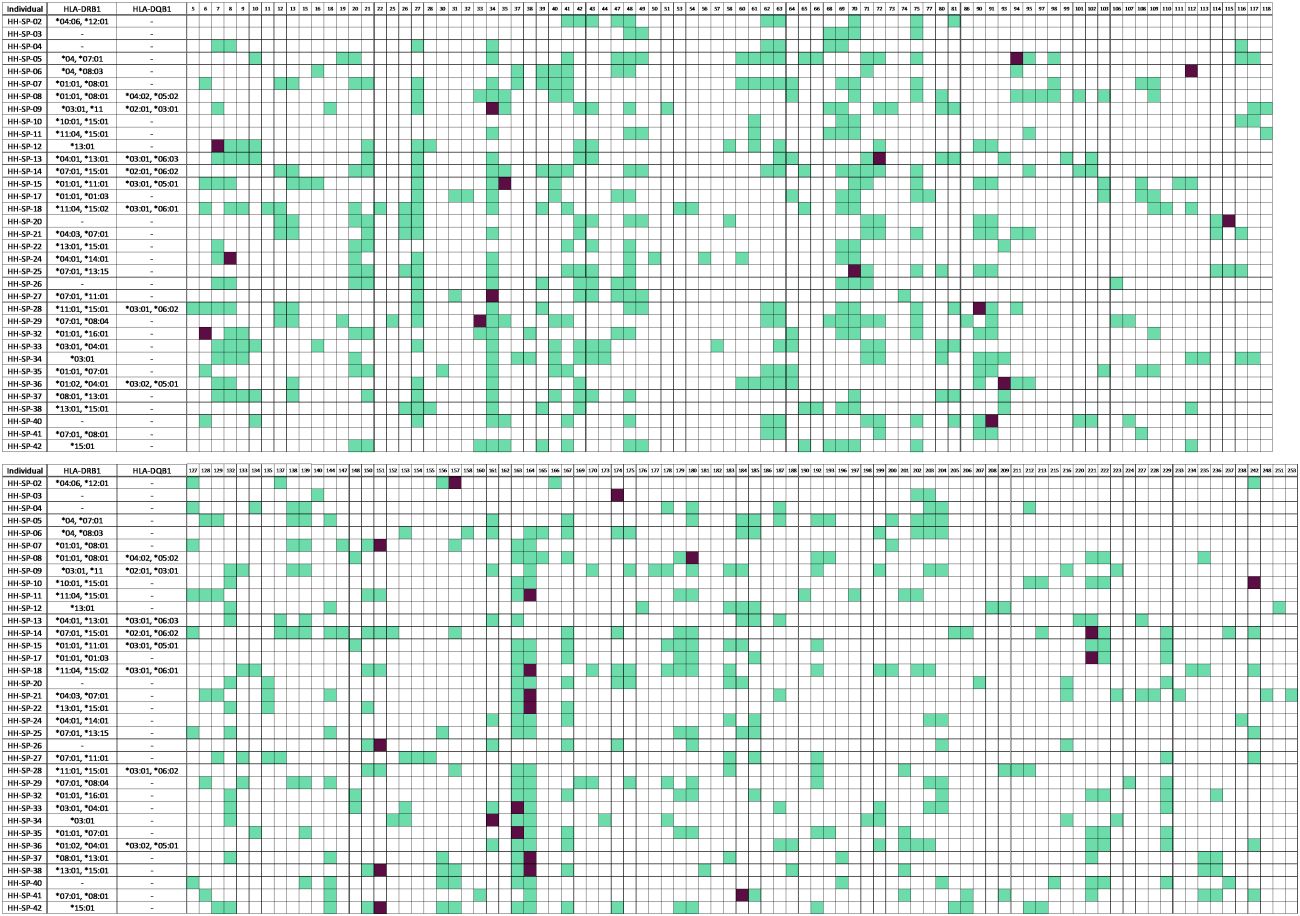
Individual CD4^+^ T‐cell response pattern to the spike glycoprotein 15‐mer peptides in the study cohort. The pattern of the CD4^+^ IFN‐γ T‐cell responses to the 15‐mer peptides of the first (upper panel) and the second half (lower panel) of the 253 15‐mer peptides. Responses are highlighted in green, and the strongest IFN‐γ response of each individual is highlighted in purple. Peptides without any response are not depicted. Bold lines indicate separate peptide pools (S1a–S6b).

The response frequency and localisation of the individual CD4^+^ T‐cell responses directed against any of the 253 peptides are depicted in Figure 3a and ranged from 0% to 80%. Figure 3b shows the 12 most broadly detected CD4^+^ T‐cell peptide specificities of our study, each with a response rate of at least 40% (14 of the 35 participants). These 12 peptides attributed to 24.1% (230 out of 955) of all CD4^+^ T‐cell responses. All patients' spike‐specific CD4^+^ T cells recognised 3 or more of these 12 most frequently detected peptide specificities. The patients recognised a median of 7 of the 12 most immunogenic peptides. Four of these peptides were located within the NTD (peptides 27, 34, 42 and 48), four in the RBD (peptides 63, 69, 70 and 75) and four outside of the functional domains (peptides 163, 164, 167 and 180). The most frequently recognised peptides for each study group varied only marginally and are shown in Supplementary figure 10. 91.4% of all study participants recognised at least one of the three most frequently recognised peptides (peptide 34 aa166–180, peptide 163 aa811–825 and peptide 164 aa816–830). The exact response pattern of each individual is depicted in Figure 4, including the peptide‐specific T‐cell response that showed the highest proportion of IFN‐γ‐producing CD4^+^ T cells for each patient. Only three of the 12 most recognised peptides were affected by mutational changes in the amino acid sequence of B.1.1.7, B.1.617.2, B.1.1.529 and its LUMs (BA.1, BA.2, BA.2.12, BA.2.75 and BA.4/5). These changes are highlighted in different colours in Figure 3 and Supplementary figure 11.

Hence, the most frequently detected peptides in our study were recognised by a large proportion of the participants despite diverse HLA backgrounds. This suggests that the presentation of these peptides can be mediated by multiple MHC class II molecules. To further prove this hypothesis, we generated HLA molecule binding data^46^ for 14 promising spike glycoprotein CD4^+^ T‐cell epitopes with a set of 11 HLA‐DRB1 molecules covering a majority of the population. Coverage of an HLA molecule was considered based on a binding affinity (IC50) of 1000 nM or lower.^47^ Additionally, we collated the results with response rates in our study cohort and *in silico*‐predicted binding data generated with the iedb.org consensus tool.^48^, ^49^

Seventy combinations of one of the 14 peptides with one of the 11 tested HLA‐DRB1 molecules showed an IC50 value < 1000 nM (Table 2) and were thus considered binding pairs. Twenty‐nine combinations were considered high‐affinity bindings because of their IC50 < 100 nM. Most of the investigated peptides showed a binding affinity < 1000 nM to multiple DRB1 alleles. For instance, peptide 48 (aa236–250) showed substantial binding affinity to nine of the 11 tested molecules (DRB1*01:01, DRB1*04:01, DRB1*04:05, DRB1*07:01, DRB1*08:02, DRB1*09:01, DRB1*11:01, DRB1*12:01 and DRB1*15:01). This supports our hypothesis of high population coverage and promiscuous MHC class II binding, and we found similar results for several highly recognised peptides (peptide 41 aa201–215, peptide 48 aa236–250, peptide 69 aa341–355, peptide 91 aa451–465 and peptide 180 aa896–910).

**Table 2:** HLA‐DRB1 binding data generated for frequently recognised spike peptide specificities. For each peptide, *in vitro*‐generated and *in silico*‐predicted DRB1 molecule binding and the response frequencies measured in our study are shown. Where available, HLA restrictions from previous studies are referenced. Bold font indicates the most likely HLA restriction.

Peptide		DRB1*01:01	DRB1*03:01	DRB1*04:01	DRB1*04:05	DRB1*07:01	DRB1*08:02	DRB1*09:01	DRB1*11:01	DRB1*12:01	DRB1*13:02	DRB1*15:01	alleles bound	other reported class II restrictions
27 CEFQFCNDPFLGVYY aa131‐145	*in vitro* binding capacity (IC50)	> 40000	> 40000	6958	4506	27456	> 40000	13478	> 40000	> 40000	> 40000	7645	0	DQB1*05:02, DQB1*05:03 [50]
	*in silico* prediction (percentile)	46	29	32	50	52	77	25	64	23	48	35		
	responding patients	4/6	2/3	4/4	‐	5/8	‐	‐	3/3	0/1	‐	3/7		
	HLA restriction references													
34 CTFEYVSQPFLMDLE aa166‐180	*in vitro* binding capacity (IC50)	**495**	5858	1152	16	**15**	1465	< 0,2	3382	416	> 40000	7244	5	DRB1*16:01, DQB1*02:01, DQB1*02:02, DQB1*05:02, DQB1*05:03 [50]
	*in silico* prediction (percentile)	**15**	64	24	9.3	**12**	59	7.8	33	40.5	43	30		
	responding patients	**6/6**	3/3	4/4	‐	**7/8**	‐	‐	3/3	0/1	‐	5/7		
	HLA restriction references					**[50]**								
41 FKIYSKHTPINLVRD aa201‐215	*in vitro* binding capacity (IC50)	64	> 40000	5252	16281	**2.7**	125	13	229	7183	9039	611	6	DRB1*13:01 [50]
	*in silico* prediction (percentile)	21	59	24	32	**4.2**	6.2	8.8	14	39	9.2	28		DRB3 [51]
	responding patients	3/6	0/3	0/4	‐	**5/8**	‐	‐	0/3	1/1	‐	3/7		
	HLA restriction references					**[51], [50]**								
48 TRFQTLLALHRSYLT aa236‐250	*in vitro* binding capacity (IC50)	38	13801	337	794	3.9	177	11	**14**	**18**	29886	**5**	9	DRB1*14:01, DQB1*05:03 [50]
	*in silico* prediction (percentile)	0.91	25	1.2	1.5	4.1	12	31	**2**	**1.79**	26	**1.2**		DRB1*04:04, DRB5 [51]
	responding patients	2/6	1/3	1/4	‐	4/8	‐	‐	**2/3**	**1/1**	‐	**5/7**		DRB1*15:02 [53]
	HLA restriction references	[52]		[52]						**[50]**		**[51], [52]**		
63 GIYQTSNFRVQPTES aa311‐325	*in vitro* binding capacity (IC50)	2392	> 40000	**62**	201	**12**	> 40000	305	1144	2072	10685	21	5	
	*in silico* prediction (percentile)	37	75	**42**	37	**17**	53	36	38	34.5	21	19		
	responding patients	2/6	1/3	**3/4**	‐	**5/8**	‐	‐	1/3	1/1	‐	2/7		
	HLA restriction references	[51]		**[51]**		**[51]**								
69 VFNATRFASVYAWNR aa341‐355	*in vitro* binding capacity (IC50)	1030	> 40000	853	976	2.7	3712	321	372	500	> 40000	**12**	7	
	*in silico* prediction (percentile)	19	58	25	13	5.3	19	18	15	31	43	**5.4**		
	responding patients	3/6	1/3	1/4	‐	2/8	‐	‐	1/3	0/1	‐	**7/7**		
	HLA restriction references													
70 RFASVYAWNRKRISN aa346‐360	*in vitro* binding capacity (IC50)	5926	> 40000	7894	6272	540	64	1380	**3.9**	31627	> 40000	**136**	4	DRB1*13:01, DRB1*14:01 [50]
	*in silico* prediction (percentile)	21	29	46	36	20	38	26	**1.2**	62.5	49	**24**		DRB5 [51]
	responding patients	5/6	0/3	1/4	‐	3/8	‐	‐	**2/3**	1/1	‐	**7/7**		
	HLA restriction references		[51]			[50]			**[51]**					
71 YAWNRKRISNCVADY aa351‐365	*in vitro* binding capacity (IC50)	22040	> 40000	**865**	3811	> 40000	852	> 40000	37	3673	12685	851	4	DRB1*13:01 [50]
	*in silico* prediction (percentile)	61	63	**68**	41	47	23	59	15	40.5	13	63		DRB4 [51]
	responding patients	1/6	2/3	**2/4**	‐	5/8	‐	‐	1/3	0/1	‐	1/7		
	HLA restriction references		[51]											
75 SASFSTFKCYGVSPT aa371‐385	*in vitro* binding capacity (IC50)	5286	> 40000	3329	559	**519**	4671	117	**626**	11462	> 40000	711	5	
	*in silico* prediction (percentile)	42	94	39	17	**18**	53	24	**40**	49	86	13		
	responding patients	5/6	0/3	0/4	‐	**5/8**	‐	‐	**2/3**	1/1	‐	3/7		
	HLA restriction references											[50]		
91 YLYRLFRKSNLKPFE aa451‐465	*in vitro* binding capacity (IC50)	7365	> 40000	590	543	575	23	13	**4.2**	323	8567	1.7	8	DRB1*11:04, DRB3 [51]
	*in silico* prediction (percentile)	22	26	17	11	3.1	5.1	13	**1.2**	5.95	11	5.3		
	responding patients	3/6	1/3	0/4	‐	4/8	‐	‐	**2/3**	0/1	‐	1/7		
	HLA restriction references								**[51]**					
163 KPSKRSFIEDLLFNK aa811‐825	*in vitro* binding capacity (IC50)	> 40000	1498	**451**	3442	1933	3414	519	**301**	3981	> 40000	6806	3	DQB1*02:02, DQB1*05:03 [50]
	*in silico* prediction (percentile)	75	19	**48**	30	59	81	61	**76**	37.5	75	70		
	responding patients	6/6	2/3	**4/4**	‐	4/8	‐	‐	**2/3**	0/1	‐	6/7		
	HLA restriction references													
164 SFIEDLLFNKVTLAD aa816‐830	*in vitro* binding capacity (IC50)	4353	3227	1098	2167	3958	15214	1298	13221	537	174	3172	2	DRB1*14:01, DQB1*05:03 [50]
	*in silico* prediction (percentile)	26	17	8.8	23	41	24	43	31	8.25	18	37		
	responding patients	6/6	3/3	3/4	‐	5/8	‐	‐	2/3	0/1	‐	6/7		
	HLA restriction references		[50]							[50]				
167 AGFIKQYGDCLGDIA aa831‐845	*in vitro* binding capacity (IC50)	1105	> 40000	836	950	740	22262	1558	3746	16980	> 40000	**716**	4	DQB1*05:03 [50]
	*in silico* prediction (percentile)	19	83	60	55	69	61	56	71	71	59	**26**		
	responding patients	5/6	0/3	2/4	‐	3/8	‐	‐	1/3	0/1	‐	**4/7**		
	HLA restriction references													
180 IPFAMQMAYRFNGIG aa896‐910	*in vitro* binding capacity (IC50)	**58**	11726	443	1608	**25**	553	7.9	23	2.2	13705	192	8	DRB1*14:01, DQB1*04:02, DQB1*05:03 [50]
	*in silico* prediction (percentile)	**3.7**	14	26	30	**26**	25	31	20	7	15	9.1		DRB1*11:04, DRB5 [51]
	responding patients	**5/6**	1/3	0/4	‐	**5/8**	‐	‐	1/3	0/1	‐	3/7		
	HLA restriction references	**[51]**							[51]	[50]		[50]		

In the case of peptide 167 (aa831–845), which showed less diverse binding than expected given its high response rate, the high recognition rate might be explicable in another way: Three of the DRB1 alleles (DRB1*04:01, DRB1*07:01 and DRB1*15:01) cover high proportions not only of our study cohort but also of a representative German population.^37^ Thus, high recognition rates of individual epitopes can either be explained by restriction to multiple molecules or restriction by a few molecules with high population coverage.

Peptide 27 (aa131–145), which elicited a peptide‐specific CD4^+^ T‐cell response in 64% of the study participants, did not show relevant binding to any DRB1 molecule tested. Likewise, *in silico* predictions revealed adjusted ranks ≥ 25 percentile, associated with non‐ or poor‐predicted binding capacity. However, this peptide was previously noted to be restricted by HLA‐DQB1*05 alleles, common in most Caucasian populations,^50^ and was found to bind HLA‐DPB1*04:01 and 04:02, the two most common DP alleles worldwide, with high affinity (data not shown).

We determined the most likely HLA‐DRB1 allele restrictions for these 14 peptides by combining the response frequencies for a corresponding allele (cut‐off > 50%) with *in vitro* binding affinities (cut‐off < 1000 nM). The most likely restrictions are indicated by the bold font in Table 2. Where available, references for certain HLA restrictions from previous studies were also included.^50^, ^51^, ^52^, ^53^

These results suggest that several peptide specificities derived from the SARS‐CoV‐2 spike glycoprotein might be promiscuous HLA binders. The match of *in vitro* HLA binding data, response frequencies from our experiments and observations from previous studies hint towards defined HLA restrictions for several of these CD4^+^ T‐cell epitopes. These data should facilitate the design of HLA–peptide tetramers, valuable reagents for use in epitope characterisation studies^35^, ^51^ and the optimisation of peptide‐based vaccines with broad population coverage.

### *Ex vivo* phenotype of infection‐primed and vaccine‐primed spike‐specific CD4 ^+^ T cells in an activation‐induced marker (AIM) assay

**Figure 5:**
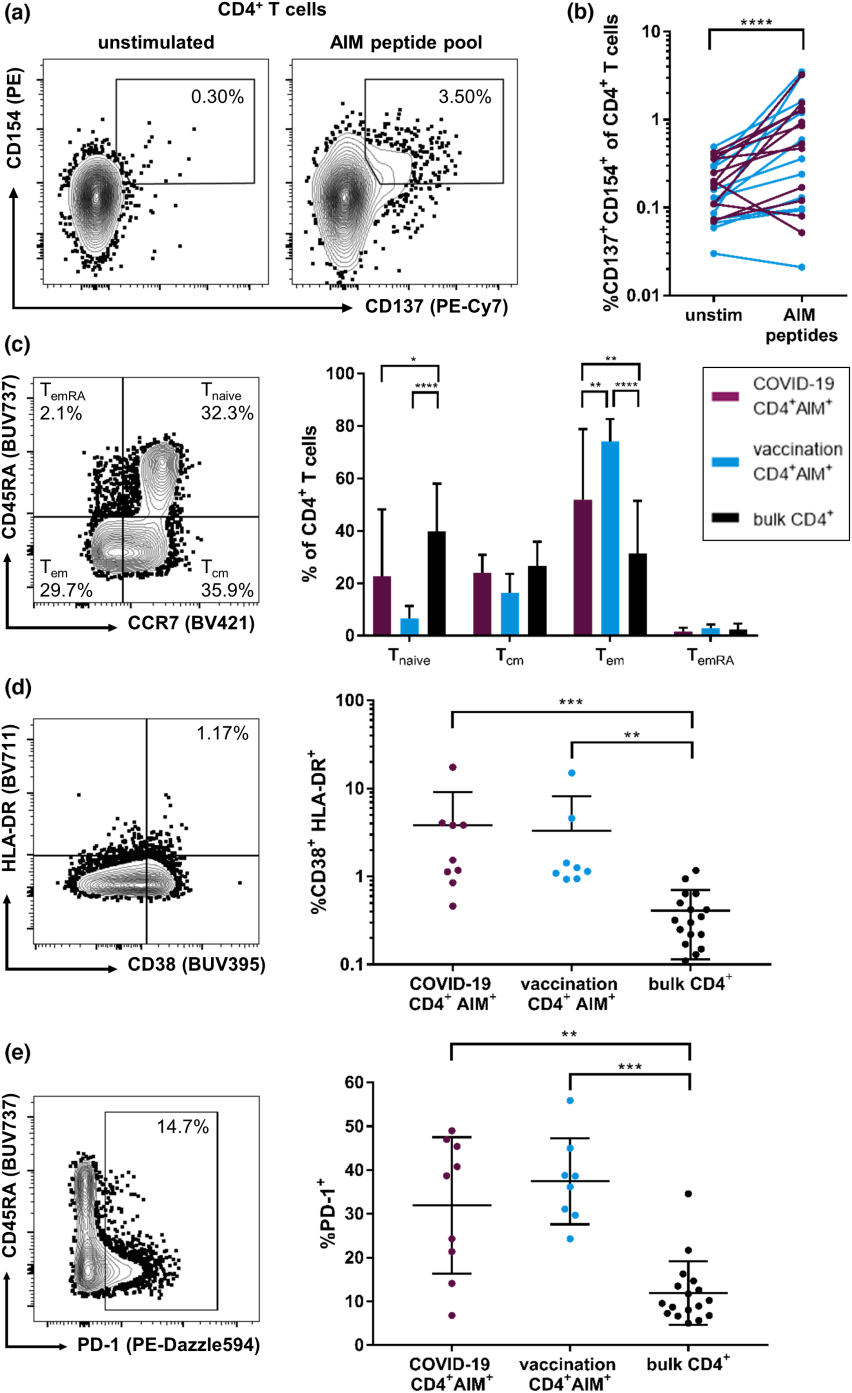
Frequencies and phenotype of AIM^+^ CD4^+^ T cells in response to a spike glycoprotein peptide pool. Thawed PBMCs were stimulated for 18 h with the peptide pool or SEB (positive control) or were left untreated (negative control) and analysed by flow cytometry. Antigen‐reactive CD4^+^ T cells were defined as CD137^+^CD154^+^ **(a)**. After stimulation with the peptides, an increase in AIM^+^ (CD137^+^CD154^+^) CD4^+^ T cells could be observed in most individuals except for a few non‐responders (SI ≤ 1.5) **(b)**. Non‐responders were excluded from further analyses. Memory phenotype of AIM^+^ CD4^+^ T cells of individuals with COVID‐19 or vaccination in comparison with bulk CD4^+^ T cells **(c)**. AIM^+^ CD4^+^ T cells of individuals with COVID‐19 and vaccinated individuals show significantly higher proportions of activation markers CD38 and HLA‐DR than bulk CD4^+^ T cells **(d)**. PD‐1 expression is increased in AIM^+^ CD4^+^ T cells of individuals with COVID‐19 and vaccination compared with that in bulk CD4^+^ T cells **(e)**. Data are expressed as mean with standard deviation. * *P* < 0.05; ** *P* < 0.01; *** *P* < 0.001; and **** *P* < 0.0001.

Using a selection of frequently detected spike‐specific peptides as specific stimulation (Supplementary table 2), we performed an activation‐induced marker (AIM) T‐cell assay similar to previously described protocols to compare the *ex vivo* magnitude and phenotype of the virus‐specific CD4^+^ T‐cell response.^54^, ^55^, ^56^, ^57^ AIM assays are believed to have a higher sensitivity than ICS, which is thought to underestimate the actual frequency of antigen‐specific T cells.^58^ Here, antigen‐reactive CD4^+^ T cells were identified by the co‐expression of CD137 (4–1BB) and CD154 (CD40L), while antigen‐reactive CD8^+^ T cells were defined as CD69^+^CD137^+^ (Figure 5a and Supplementary figure 12a).

We could observe a significant increase (indicated by a stimulation index, SI, of ≥ 1.5) of AIM^+^ CD4^+^ and CD8^+^ T cells after stimulation with the peptide pool in the samples of *n* = 9 individuals with COVID‐19 and *n* = 8 individuals after vaccination (Figure 5b and Supplementary figure 12b). Neither frequencies nor stimulation indices of AIM^+^ CD4^+^ T cells significantly differed between vaccinated and infected individuals (data not shown). Three individuals in each group did not show an SI ≥ 1.5 in AIM^+^ T cells after peptide stimulation. These non‐responders were excluded from the further phenotypic analyses of the antigen‐reactive T cells. In the remaining subjects, most AIM^+^ CD4^+^ T cells showed an effector memory (*T*_em_; CD45RA^−^CCR7^−^) phenotype (Figure 5c). Between infected and vaccinated individuals, CD4^+^ AIM^+^ T cells showed similar memory distribution; only the proportion of *T*_em_ cells of spike‐reactive CD4^+^ T cells was increased in vaccinated individuals compared with that in COVID‐19 patients (*P* = 0.0049).

We further assessed the phenotype of AIM^+^ CD4^+^ T cells. The antigen‐reactive CD4^+^ T cells of individuals with COVID‐19 were significantly more activated (indicated by co‐expression of CD38 and HLA‐DR; Figure 5d) and showed increased frequencies of PD‐1^+^, CD57^+^, TIGIT^+^ and KLRG1^+^ cells than bulk CD4^+^ T cells, while the CD127^+^ frequency was reduced (Figure 5e and Supplementary figure 13a, d). For most of the markers assessed, the phenotype of AIM^+^ CD4^+^ T cells from vaccinated individuals resembled the phenotype of antigen‐reactive CD4^+^ T cells from COVID‐19 subjects. The phenotype of spike peptide pool‐reactive CD8^+^ T cells showed only minor differences compared to the corresponding AIM^+^ virus‐specific CD4^+^ T cells (Supplementary figure 12c–i).

In summary, the peptide pool containing frequently detected epitopes in our study elicited substantial *ex vivo* CD4^+^ T‐cell responses in 17 of 23 individuals. Phenotypically, spike‐reactive CD4^+^ T cells exhibited an effector memory phenotype and showed only minor differences between infected and vaccinated individuals.

## Discussion

The main goal of this study was to comprehensively investigate the spike‐specific CD4^+^ T‐cell response at high resolution on a single peptide level in a large cohort of individuals with known HLA backgrounds. Furthermore, we explored potential differences and similarities in the breadth, distribution, magnitude and phenotype of CD4^+^ T‐cell responses directed against the SARS‐CoV‐2 spike glycoprotein between vaccine recipients, COVID‐19 patients and patients who experienced both ways of immunisation.

Through the sensitive and well‐established^35^, ^38^, ^39^, ^40^, ^41^, ^42^ two‐step approach of combining an IFN‐γ ELISpot with intracellular staining for IFN‐γ, we detected a median of 29 (range 10–45) spike peptide‐specific CD4^+^ T‐cell responses. Overall, we saw a comparable breadth of the spike‐specific CD4^+^ T‐cell response between SARS‐CoV‐2 infected patients, uninfected vaccinees and a group with mixed immunisation. Importantly, this shows that the vaccination‐induced spike‐specific T‐cell immunity is not significantly different or inferior to that generated following SARS‐CoV‐2 infection and that vaccination against SARS‐CoV‐2 primes CD4^+^ T‐cell responses directed at a broad range of peptide specificities within the spike glycoprotein.

With our *in vitro* study design, we could detect the highest percentage of IFN‐γ^+^ CD4^+^ T cells in the COVID‐19 group (median 0.35% IFN‐γ^+^ of CD4^+^ T cells) and the lowest in participants who had only received two vaccination doses (median 0.091% IFN‐γ^+^ of CD4^+^ T cells). Patients after three vaccinations showed higher response magnitudes of the spike‐specific CD4^+^ T‐cell response (median 0.175% IFN‐γ^+^ of CD4^+^ T cells). This demonstrates that additional booster vaccinations may not necessarily increase the number of spike‐specific CD4^+^ T‐cell peptide specificities targeted but rather the percentage of CD4^+^ T cells reacting to these specificities. Likewise, others could show increased cytokine responses after administration of a third vaccination.^29^, ^59^ A meta‐analysis could reveal differences between distinct vaccination regimens with regard to not only T‐cell and antibody immunity but also clinical endpoints.^60^ We could not replicate these findings, but the validity remains elusive because of relatively low case numbers for every vaccination regime in our cohort. Generally, as a result of real‐life sampling in a tertiary care hospital, the current cohort was heterogeneous with regard to the applied vaccination regime. Therefore, the data set was too small to conduct more detailed analyses of particular subpopulations.

To account for differences between the patient groups, we investigated correlations of the T‐cell response with the time since the last immunising event. Interestingly, neither the number of spike‐specific CD4^+^ T‐cell responses nor the magnitude of these responses was significantly impacted by the time after the last immunising event. The time since the last immunising event ranges in our cohort from 1 day to 448 days (median 40 days), which hints towards a SARS‐CoV‐2 spike‐specific T‐cell response that is established early on during the infection with SARS‐CoV‐2 or after vaccination and is sustained for at least several months. Overall, our research suggests that while there is a decline in antibody titres, the memory T cells should render longer‐lasting protective effects. This notion is supported by other groups, who also observed long‐lasting T‐cell responses.^61^, ^62^

Three individuals in our cohort, HH‐SP‐02, HH‐SP‐04 and HH‐SP‐05, were immunosuppressed because they either received immunosuppressive medication or chemotherapy. Despite a dysfunctional B‐cell response indicated by a negative nucleocapsid and spike antibody titres for HH‐SP‐02 and HH‐SP‐04, they showed substantial spike‐specific CD4^+^ T‐cell responses with 16 and 19 recognised peptide specificities, respectively. This is in line with previous studies that could show sustained T‐cell responses in a B‐cell‐depleted patient.^63^, ^64^ Generally, immunosuppression is not associated with an increased risk for severe COVID‐19 but rather with viral persistence and escape mutations^65^, ^66^, ^67^, ^68^, ^69^ and further studies need to determine the spike‐specific T‐cell response in more heavily immunosuppressed individuals.

Despite statistically non‐significant differences, the specificities within the RBD were the most broadly recognised of all functional domains. Every patient showed at least one response to a peptide located within the RBD. Likewise, a previous study reported high immunogenicity of the RBD^70^ and another one could show that strong spike‐ and especially RBD‐specific circulating T follicular helper cells correlate with the maintenance of humoral immunity.^71^ In addition to the already known protective aspect of neutralising anti‐RBD antibodies,^20^ these findings support the upcoming efforts to develop a vaccine based on the SARS‐CoV‐2 spike RBD.^72^, ^73^, ^74^ Such a vaccine could likely induce protective, spike‐specific CD4^+^ T‐cell responses in a substantial proportion of patients.

In line with this idea, we found 12 highly immunogenic peptides that each had a response frequency of 40% or higher, four of which are located in the RBD (peptides 63, 69, 70 and 75). These 12 peptide specificities are mostly consistent between the different ways of immunisation (Supplementary figure 11) and are reflected by previously performed experiments: Grifoni *et al*. predicted the sequences of peptides 48, 69 and 180 to be immunogenic targets.^32^ These peptides were broadly recognised in our experiments. Furthermore, a highly conserved and immunodominant spike‐specific CD4^+^ T‐cell epitope spanning the amino acids 346–365 was previously reported.^70^ We can confirm this highly immunogenic region with 91.4% of individuals showing at least one CD4^+^ T‐cell response to one of the five peptides (peptides 68–72) containing major parts of this sequence. Peptides 69 (aa341–355) and 70 (aa346–360) within this region belong to the most frequently detected peptides in our study.

Previous studies identified a highly cross‐clade conserved region near the fusion peptide (aa816–830) producing cross‐reactive T‐cell clones *in vitro*, which recognise the spike proteins of six different coronaviruses including four common cold coronaviruses (CCC; OC43, HKU1, NL63 and 229E), SARS‐CoV and SARS‐CoV‐2.^70^, ^75^ The peptides 163–165 (aa811–835) cover this sequence, and the peptide specificities 163 (recognition rate 68.57%) and 164 (recognition rate 74.29%) belong to the three most immunogenic specificities in our study. Of note, eight subjects showed the highest proportion of IFN‐γ^+^ CD4^+^ T cells in response to either of these peptides (Figure 4). It has been repeatedly reported that a substantial number of CD4^+^ T cells specific to epitopes of the spike glycoprotein pre‐exist in unexposed individuals.^51^, ^54^, ^75^, ^76^, ^77^, ^78^, ^79^ This might be because of sequence homologies between the SARS‐CoV‐2 spike glycoprotein and proteins of other human coronaviruses. For 15 of the 35 study participants, we measured antibody responses against CCC using a commercially available line blot.^35^ Of these 15 study participants, five showed a positive result for either OC43, HKU1, NL63 or 229E. The prevalence of CCC antibody responses could not be associated with a CD4^+^ T‐cell response to either of the peptides 163–165 or its magnitude and vice versa. Our preliminary data suggest that HLA restriction for these peptide specificities seems to be of higher importance than exposure to CCC for recognition of this region. Further studies uncovering the role of cross‐priming of CCC‐specific and SARS‐CoV‐2 immune responses are warranted.^51^, ^54^, ^70^, ^75^, ^76^, ^77^, ^78^, ^79^, ^80^

*In vitro* HLA‐binding experiments revealed promiscuous binding and high potential population coverage for the majority of the most immunogenic epitopes. Here, we report peptide specificities with broad estimated HLA coverage that could be used to assess T‐cell immunity even in diverse populations. We used a selection of these frequently detected peptides to compare the *ex vivo* spike‐specific T‐cell response of vaccinees and COVID‐19 patients. We did not find differences with regard to the frequency or phenotype of the vaccine‐ versus infection‐induced antigen‐specific T cells. These types of analyses have to be extended to larger cohorts and more extensive phenotypic and cytokine examination. Also, other marker combinations for AIM assays could lead to deviating results.^81^, ^82^, ^83^ The assessment of cytokine production in response to the peptide pool would allow assumptions with regard to the T‐cell (poly‐) functionality.^28^, ^84^, ^85^ Most importantly, the assessment of cellular immunity towards SARS‐CoV‐2 using these peptides in combination with the antibody status could provide a better understanding of the adaptive immune response and facilitate more refined public health interventions.^86^

Other groups could show that the T‐cell and antibody responses against SARS‐CoV‐2 spike glycoprotein remain effective against the currently dominating B.1.1.529 VoC.^45^, ^87^, ^88^, ^89^, ^90^ We support these observations in this high‐resolution analysis. We found that every individual in our study still had at least seven CD4^+^ T‐cell specificities that were conserved in all VoCs and LUMs investigated. Additionally, most of the most frequently detected specificities are conserved in the B.1.1.529 VoC. Of the group of the 12 most frequently detected peptides, only three were affected by mutational changes in B.1.1.529: peptide 27 (aa131–145, NTD), peptide 42 (aa206–220, NTD) and peptide 75 (aa371–385, RBD) are changed in LUMs BA.1, BA.2, BA.2.12, BA.4/5 and BA.2.75. The mutations in other previously circulating VoCs affect the same peptides. Since nine of the 12 peptides remain unchanged in all VoCs that have emerged during the course of the COVID‐19 pandemic, this hints towards a broader cross‐variant reactivity of the COVID‐19‐infection‐ and vaccine‐induced T‐cell response. Future studies should assess the degree of cross‐reactivity of antigen‐specific T cells towards mutated epitopes. This research could be helpful to predict the establishment and sustainment of protective immunity against future virus variants. Indeed, the spike‐specific CD8^+^ T‐cell response seems to be more affected by viral escape mutations^91^, ^92^ but was not the focus of this study.

As already noted, our study has several limitations, the biggest of which being the *in vitro* design of the main assays, which to some extent limits the comparability of our results to the conditions *in vivo*. However, the results of the *ex vivo* AIM assay conducted within this study largely support the *in vitro* results. Potentially, our results are biased by the study design in which we used 15‐mer peptides that are more likely to bind to HLA class II molecules than to class I molecules,^93^ which might lead to reduced detection of CD8^+^ T‐cell specificities.

This most detailed investigation into single‐peptide T‐cell responses, which also provides the HLA background of most participants, demonstrates how both COVID‐19 infection and vaccination against SARS‐CoV‐2 produce a broadly directed T‐cell immunity directed throughout the spike glycoprotein. This comprehensive high‐resolution analysis of immunodominant peptide specificities each covering a large population will be an essential data set in the investigation of spike‐specific T‐cell responses.

## Methods

### Patient cohort

All individuals were recruited at the University Medical Center Hamburg‐Eppendorf. Unvaccinated individuals with COVID‐19 (*n* = 8) and individuals with a breakthrough infection (*n* = 5) were hospitalised with COVID‐19. Uninfected individuals with vaccination (*n* = 16) and previously infected individuals, who received a subsequent vaccination (*n* = 6), were recruited among the medical and non‐medical staff of the University Medical Center Hamburg‐Eppendorf and associated institutions. All study participants gave written informed consent. The study was approved by the local ethics board of the *Ärztekammer Hamburg* (PV4780 and PV7298).

For infected individuals, infection with SARS‐CoV‐2 was confirmed by polymerase chain reaction (PCR) from oropharyngeal and/or nasopharyngeal swabs as previously described.^33^ Anti‐SARS‐CoV‐2 spike and nucleoprotein antibody titres were determined using the DiaSorin LIAISON (anti‐S‐trimer) (DiaSorin, Saluggia, Italy) and the Roche Elecsys (anti‐S RBD) (Roche Diagnostics, Basel, Switzerland) immunoassays as previously described.^57^, ^94^ For patients with sufficient cell counts, HLA typing from whole blood was performed at the Institute of Transfusion Medicine at the University Medical Center Hamburg‐Eppendorf using PCR‐SSO (One Lambda, Canoga Park, CA, USA) technology as previously described.^95^

### Sample processing and T‐cell expansion

Venous whole‐blood samples from the study participants were collected in Vacutainer CPTs (BD, Franklin Lakes, NJ, USA). Peripheral blood mononuclear cells (PBMCs) were isolated by centrifugation and used freshly. PBMCs were resuspended in Roswell Park Memorial Institute medium (RPMI 1640; Gibco, Thermo Fisher Scientific, Waltham, USA) supplemented with 10% fetal calf serum (FCS), 1% penicillin and streptomycin, and 1% HEPES buffer (Gibco, Thermo Fisher Scientific). The antigen‐specific multiclonal T‐cell expansion was induced by stimulation with each of the 12 peptide pools consisting of 21 or 22 peptides of the overall 253 overlapping 15‐mer peptides covering the whole SARS‐CoV‐2 spike glycoprotein (Supplementary table 2). To provide costimulatory signals, anti‐CD28/anti‐CD49d antibodies (BD) and 50 U mL^−1^ rIL‐2 (Miltenyi Biotec, Bergisch‐Gladbach, Germany) were added to the cell culture medium. After 14 days, the cells were harvested and used for the T‐cell assays described below.

### IFN‐γ ELISpot assay

IFN‐γ ELISpot assays were performed as described.^35^, ^38^, ^39^, ^40^, ^41^, ^42^ In short, approximately 50 000 pre‐cultured cells per well were plated into 96‐well plates pre‐coated with IFN‐γ antibodies (clone 1‐D1K; Mabtech AB, Nacka Strand, Sweden). The cells were then individually stimulated with each of the 21 (Pools 1A–6A) or 22 (Pool 6B) peptides (synthesised by peptides & elephants GmbH, Hennigsdorf, Germany) of the corresponding peptide pool at a concentration of 10 μg mL^−1^ in medium overnight at 37°C and 5% CO_2_. Anti‐CD3 antibody‐stimulated cells served as positive control and unstimulated cells as negative control.

IFN‐γ was detected with a biotinylated anti‐IFN‐γ antibody (clone 7‐B6‐1; Mabtech AB), which was incubated with alkaline phosphatase‐conjugated streptavidin (Streptavidin‐ALP) and 5‐bromo‐4‐chloro‐3‐indolyl phosphate (BCIP)/nitroblue tetrazolium (NBT) substrate solution. Results were considered positive if the response well showed at least three times the number of IFN‐γ‐spots compared with the negative control well. Positive results were verified and differentiated for CD4^+^ or CD8^+^ response by flow cytometric and intracellular cytokine staining (ICS) for IFN‐γ.

### Intracellular cytokine staining

Positive results in the ELISpot assay were validated by ICS for IFN‐γ as described previously.^35^ The pre‐cultured cells were restimulated with the peptides showing a positive result at a concentration of 10 μg mL^−1^ for 16 h at 37°C and 5% CO_2_. After one hour, Brefeldin A (Sigma‐Aldrich, St. Louis, MO, USA) in a final concentration of 5 μg mL^−1^ was added to inhibit cytokine secretion.

The cells were stained with Zombie NIR fixable viability dye (BioLegend, San Diego, CA, USA) and the following fluorochrome‐conjugated monoclonal antibodies on the cell surface: anti‐CD3 (clone UCHT1, Alexa Fluor 700), anti‐CD4 (clone SK3, BV510), anti‐CD8 (clone RPA‐T8, PerCP‐Cy5.5), anti‐CD14 (clone 63D3, APC‐Cy7) and anti‐CD19 (clone HIB19, APC‐Cy7). After fixation and permeabilisation using the FoxP3 transcription factor staining buffer set (eBioscience, Thermo Fisher Scientific), the cells were stained for intracellular IFN‐γ using a monoclonal anti‐IFN‐γ antibody (clone 4S.B3, PE‐Dazzle594). All antibodies were purchased from BioLegend. The cells were acquired on a LSRFortessa II cytometer (BD) using FACSDiva version 8 for Windows (BD). The full gating strategy is reproduced in Supplementary figure 14.

### *In vitro* HLA binding assays and *in silico* predictions

*In vitro* binding assays with 14 of the peptides that elicited a spike‐specific CD4^+^ T‐cell response were performed using purified HLA class II molecules, as previously described.^46^ Coverage of an allele was considered based on a corresponding binding affinity (IC50) of 1000 nM or lower.

*In silico* MHCII binding predictions were made using the IEDB analysis resource Consensus tool.^48^, ^49^

### Activation‐induced marker (AIM) assay and *ex vivo* immunophenotyping

The AIM assay was performed as previously described^54^, ^55^, ^56^, ^57^ with a few adaptations. In short, cryopreserved PBMCs were stimulated for 18 h with the pool of 11 frequently recognised peptides (Supplementary table 2) or staphylococcal enterotoxin B (SEB) (Sigma‐Aldrich) or were left untreated. The cells were then washed and stained with Zombie NIR fixable viability dye (BioLegend) and fluorochrome‐labelled monoclonal antibodies targeting CD45RA (clone HI100, BUV737; BD), CD38 (clone HB7, BUV395; BD), CD4 (clone RPA‐T4, BV785), HLA‐DR (clone L243, BV711), CD8 (clone RPA‐T8, BV650), TIGIT (clone A15153G, BV605), CD57 (clone QA17A04, BV510), CCR7 (clone G043H7, BV421), KLRG1 (clone SA231A2, FITC), CD127 (clone A019D5, PerCP‐Cy5.5), CD137 (clone 4B4‐1, PE‐Cy7), PD‐1 (clone EH12.2H7, PE‐Dazzle594), CD3 (clone UCHT1, Alexa Fluor 700), CD69 (clone FN50, APC), CD14 (clone 63D3, APC‐Cy7) and CD19 (clone HIB19, APC‐Cy7). After fixation and permeabilisation (FoxP3 transcription factor staining buffer kit; eBioscience), the cells were stained for intracellular CD154 (clone 24–31, PE). If not stated otherwise, all monoclonal antibodies were purchased from BioLegend.

### Data analysis and statistics

The analysis of flow cytometric data was performed in FlowJo version 10 (FlowJo LLC, Ashland, OR, USA) for Windows. All graphs and statistics were created in GraphPad Prism version 7 (GraphPad Software Inc., San Diego, CA, USA) for Windows. Data are visualised as mean with standard deviation. The following tests for statistical significance were used: the Mann–Whitney *U*‐test (for testing of two groups); Kruskal–Wallis and ANOVA with Dunn's correction for multiple analyses (for testing of three or more groups); the Wilcoxon matched‐pair test (for paired testing); and non‐parametric Spearman's correlation (for correlation analysis).

The stimulation index (SI) for the AIM assay was calculated as the quotient of the frequencies of AIM‐positive cells in the stimulated and unstimulated sample. A SI ≥ 1.5 was defined as a positive response.

For all tests, two‐tailed *P*‐values were generated and results with a *P*‐value < 0.05 were considered statistically significant. Levels of significance are translated to asterisks as follows: *P < 0.05; **P < 0.01; ***P < 0.001; and ****P < 0.0001.

## AUTHOR CONTRIBUTIONS

**Hendrik Karsten:** Conceptualization; data curation; formal analysis; investigation; methodology; visualization; writing – original draft; writing – review and editing. **Leon Cords:** Conceptualization; data curation; formal analysis; investigation; methodology; visualization; writing – original draft; writing – review and editing. **Tim Westphal:** Data curation; formal analysis; investigation; methodology. **Maximilian Knapp:** Data curation; investigation; visualization. **Thomas Theo Brehm:** Data curation; resources. **Lennart Hermanussen:** Resources. **Till Frederik Omansen:** Resources. **Stefan Schmiedel:** Resources. **Robin Woost:** Investigation; project administration. **Vanessa Ditt:** Resources. **Sven Peine:** Resources. **Marc Ltgehetmann:** Resources. **Samuel Huber:** Resources; writing – review and editing. **Christin Ackermann:** Resources; supervision; writing – review and editing. **Melanie Wittner:** Resources; supervision; writing – review and editing. **Marylyn Martina Addo:** Resources. **Alex Sette:** Resources; validation. **John Sidney:** Resources; validation. **Julian Schulze zur Wiesch:** Conceptualization; funding acquisition; methodology; project administration; resources; supervision; writing – original draft; writing – review and editing.

## Conflict of interest

The authors do not report any competing interests.

## Supporting information


Supplementary figures 1‐14

Supplementary table 1

Supplementary table 2
