# Supplementary material for: High‐resolution analysis of individual spike peptide‐specific CD4 + T‐cell responses in vaccine recipients and COVID‐19 patients
Source: Clin Transl Immunology. 2022 Aug 9;11(8):e1410. doi: 10.1002/cti2.1410 (PMC9363231; doi:10.1002/cti2.1410)
Supplement: Supplementary file 1 — Supplementary figures 1‐14 [file CTI2-11-0-s003.pdf]

Karsten, Cords *et al.*
Spike peptide-specific $CD4^+$ T cells

SUPPLEMENTARY FIGURES 1-14

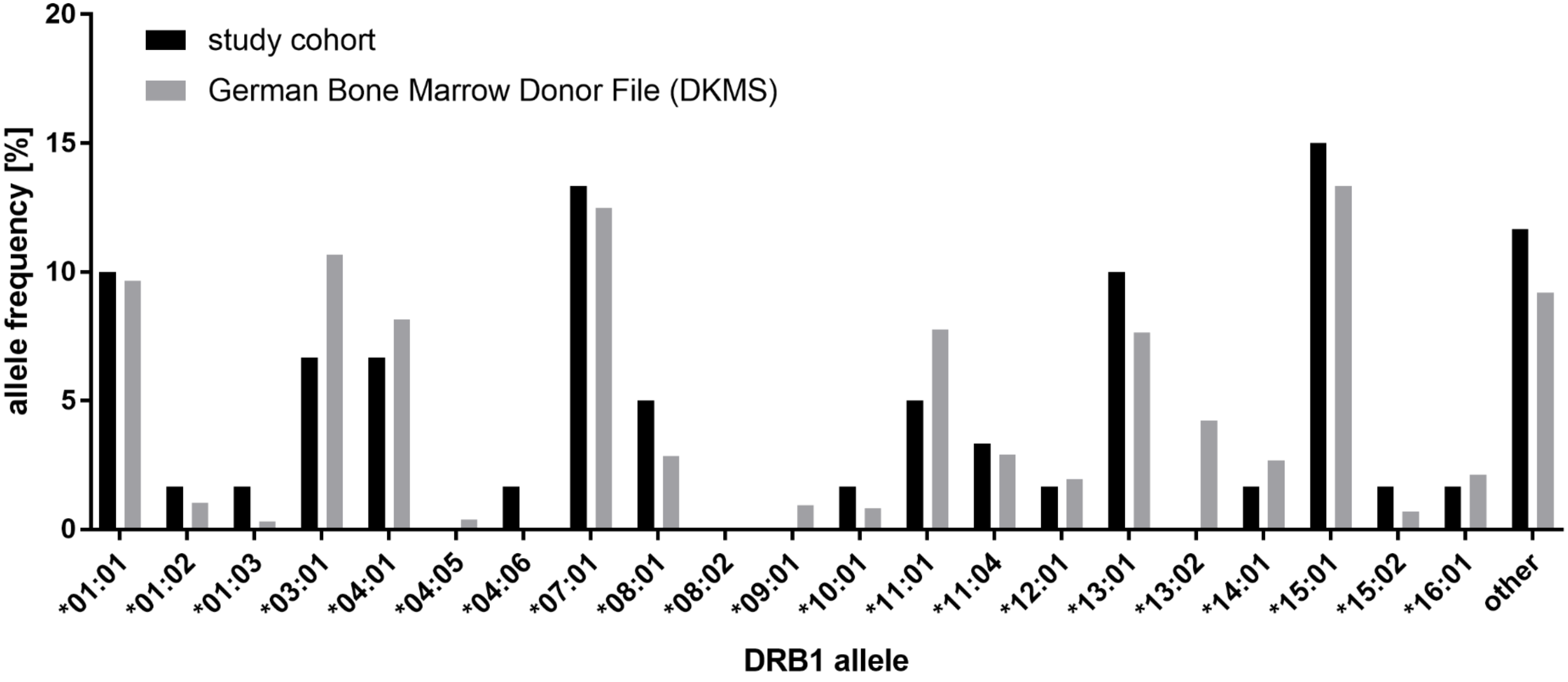

Supplementary Figure 1. HLA-DRB1 allele frequencies in the study cohort (black) and a representative German population (grey, German Bone Marrow Donor File, n=3456066). Allele frequency is defined as the total number of copies of the allele in the population sample (alleles / 2n).

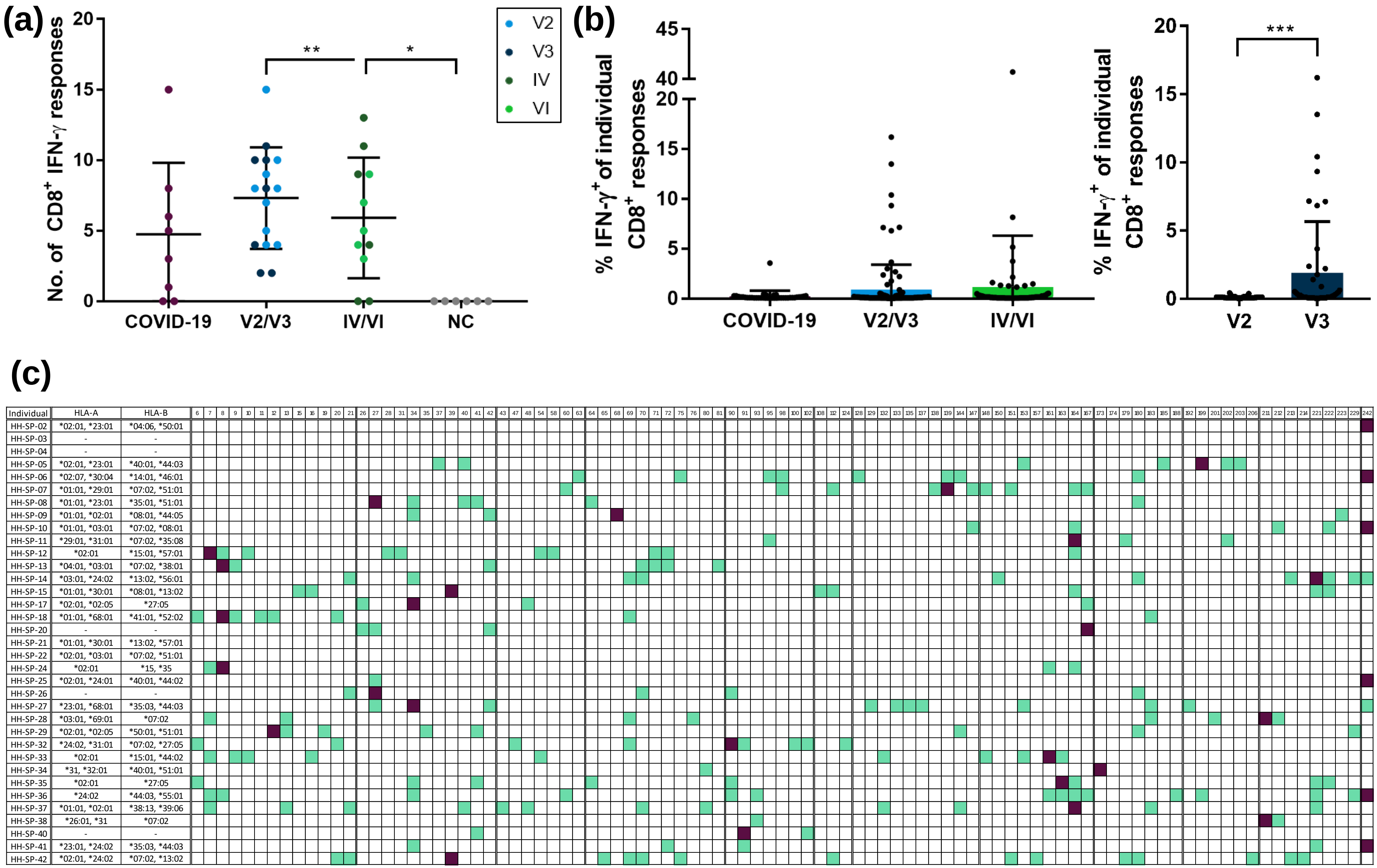

Supplementary Figure 2. CD8⁺ IFN-γ T-cell responses of individuals with COVID-19, vaccination or a combination of both to SARS-CoV-2 spike glycoprotein peptides. The number of spike peptide-specific CD8⁺ T-cell responses **(a)** and the magnitude of the individual responses **(b)** are compared between the study groups. The pattern of the CD8⁺ T-cell responses to the 253 15-mer peptides are depicted **(c)**. Responses are highlighted in green, the strongest CD8⁺ T-cell response of each individual is highlighted in purple. Peptides without any CD8⁺ T-cell response are not depicted. Bold lines indicate separate peptide pools (S1a-S6b).

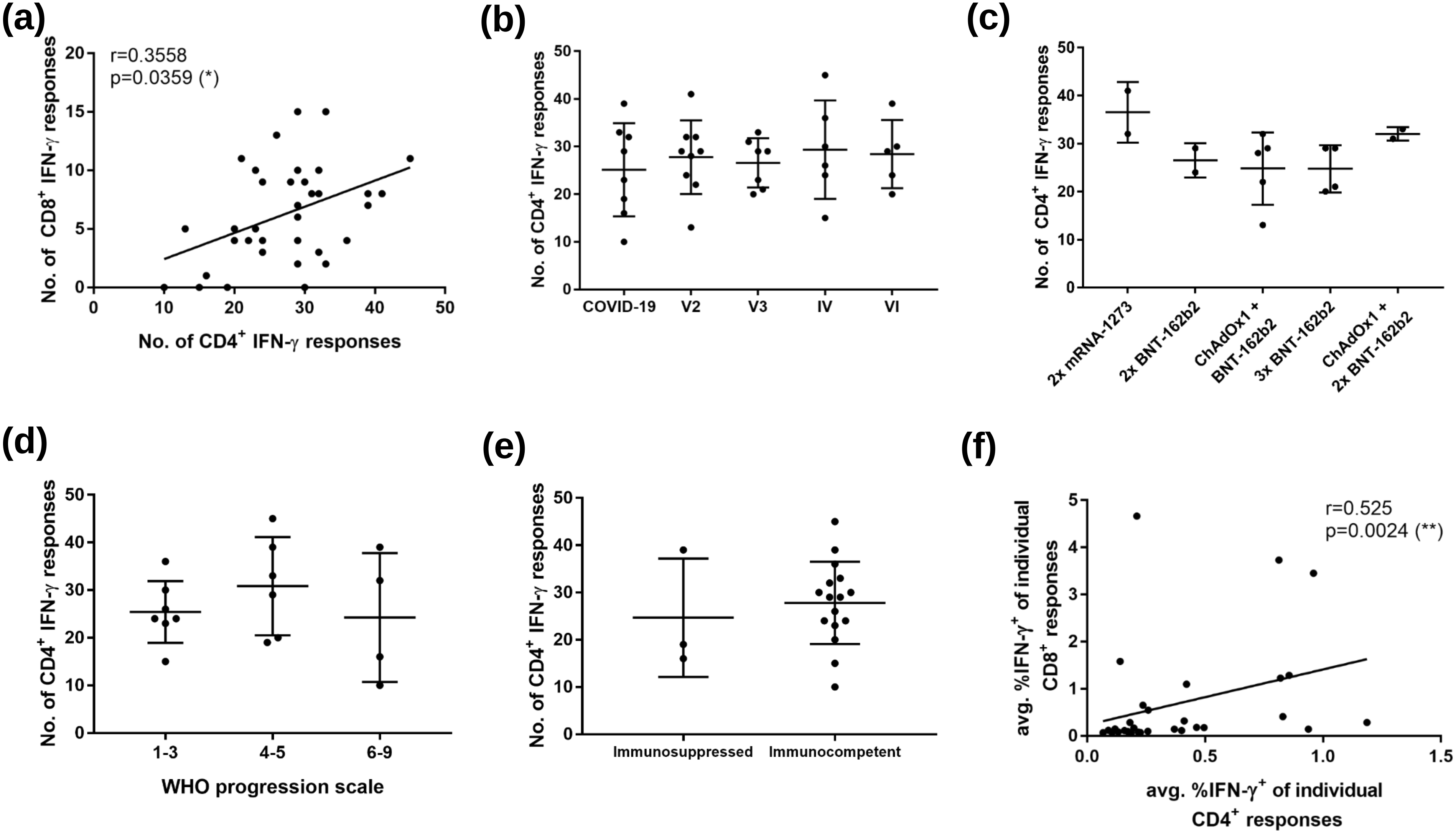

Supplementary Figure 3. Sub-analysis of number and magnitude of $CD4^+$ and $CD8^+$ T-cell responses of the individual study participants

The number **(a)** of the $CD4^+$ and $CD8^+$ T-cell responses for each individual correlate significantly. Each dot represents one study participant. More detailed subdividing of the study participants by study groups **(b)**, by vaccination regime **(c)**, disease severity **(d)** and state of immunocompetence **(e)** does not reveal significant differences in the number of $CD4^+$ T-cell responses. The average magnitude **(f)** of the $CD4^+$ and $CD8^+$ T-cell responses for each individual correlate significantly.

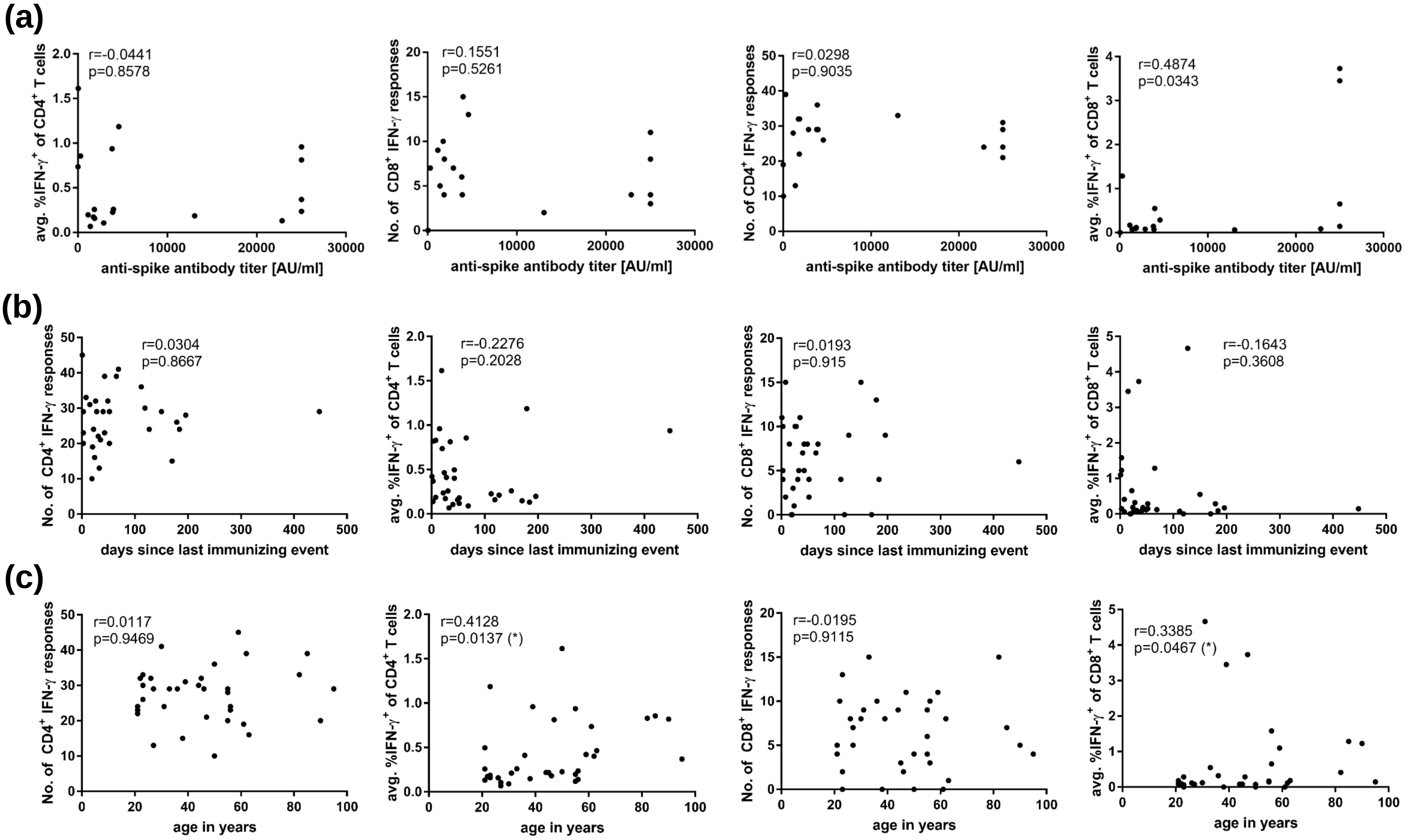

Supplementary Figure 4. Correlation analysis of number and magnitude of $CD4^+$ and $CD8^+$ T-cell responses with anti-spike antibody titer (a), time since the last immunizing event in days (b), and age in years (c).

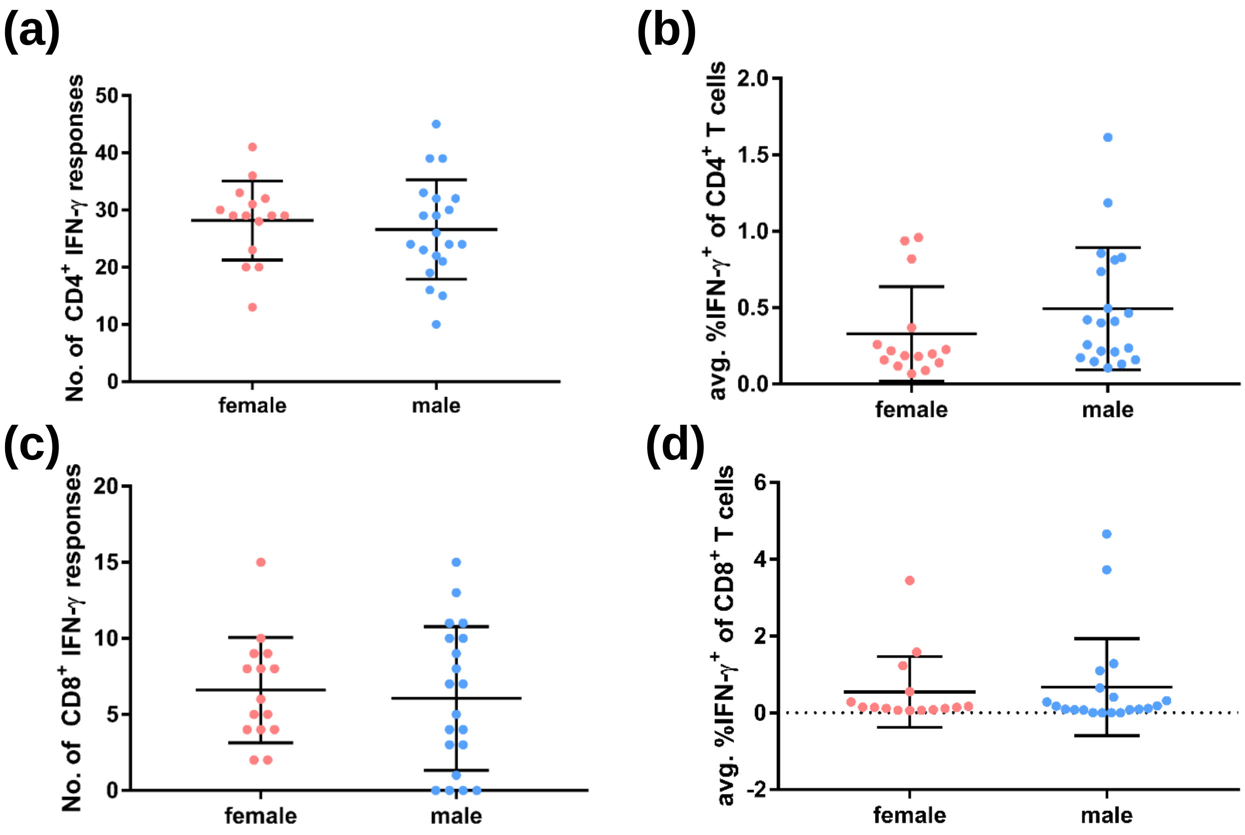

Supplementary Figure 5. Analysis of number and magnitude of $CD4^+$ and $CD8^+$ T-cell responses according to the sex at birth.
There are no significant differences on the number and magnitude of $CD4^+$ T-cell responses **(a-b)** and $CD8^+$ T-cell responses **(c-d)** between individuals with female or male sex.

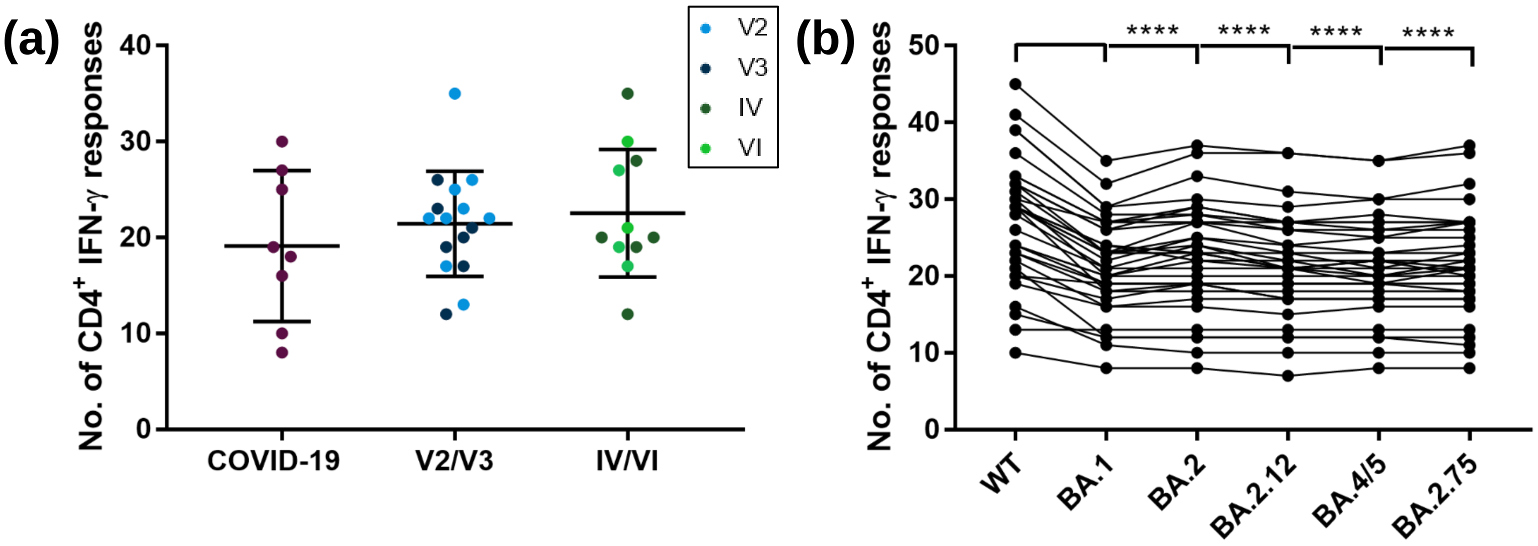

Supplementary Figure 6. The number of spike-specific CD4⁺ T-cell responses with consideration of mutational changes in the Omicron VoCs and different ways of immunization. The number of CD4⁺ T-cell responses for each study participant without peptides that contain mutational changes in the B.1.1.529.4/5 VoC **(a)**. After the exclusion of peptides affected in different variants, the number of responses is significantly reduced compared to all peptides **(b)**. Each group of connected dots represents one study participant. The displayed level of significance is calculated in comparison to the wild-type peptide sequence.

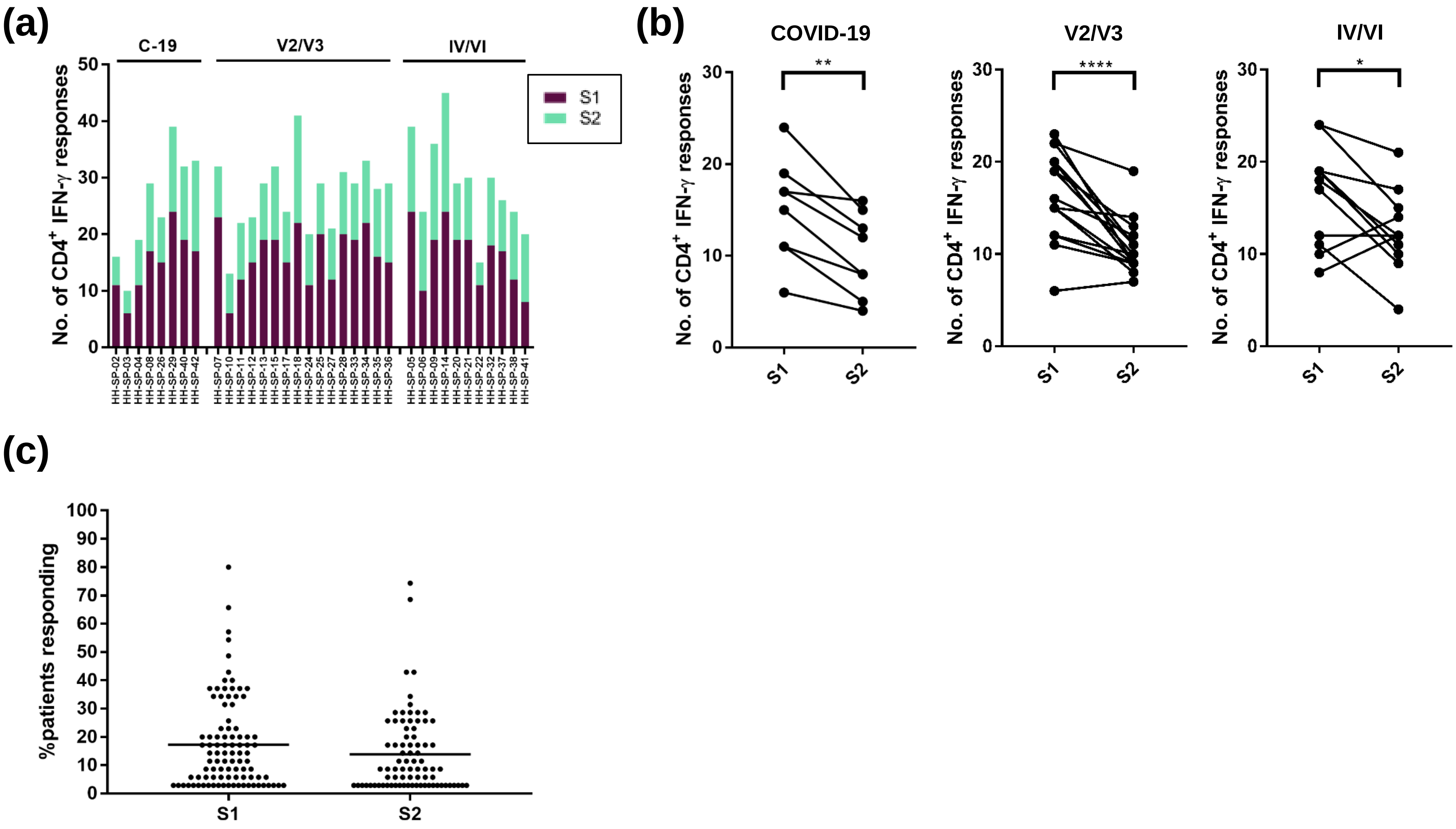

Supplementary Figure 7. Response pattern in the S1 and S2 subunits. Number of $CD4^+$ T-cell responses (S1 purple, S2 green).**(a)**. The study participants recognized significantly lower numbers of peptides located in the S2 subunit compared to the S1 subunit **(b)**. This was still true when adjusting for the different lengths of the subunits. Peptides in the S1 subunit that elicited $CD4^+$ T-cell responses in our study were on average recognized by slightly more patients compared to recognized peptides from the S2 subunit **(c)**.

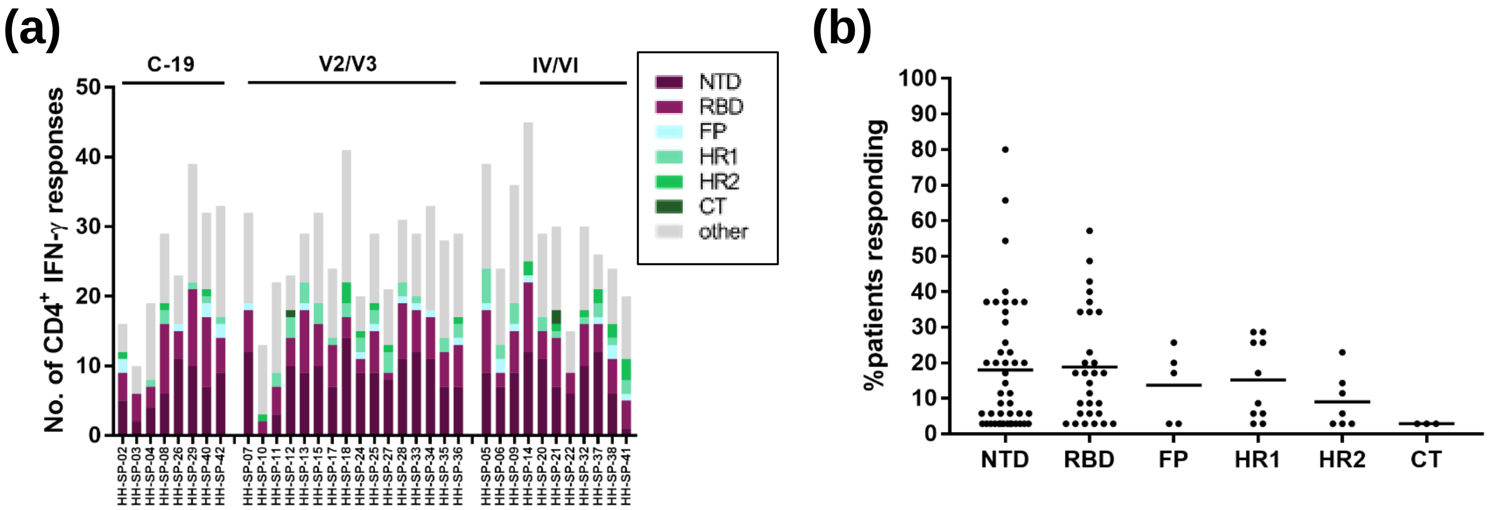

Supplementary Figure 8. Response pattern in the functional domains. Number of CD4+ T-cell responses of each patient expressed as the relative share of responses to peptides of the different functional domains. **(a)**. Peptides in the RBD that elicited CD4+ T-cell responses in our study were on average recognized by slightly more patients compared to recognized peptides from the other functional domains **(b)**.

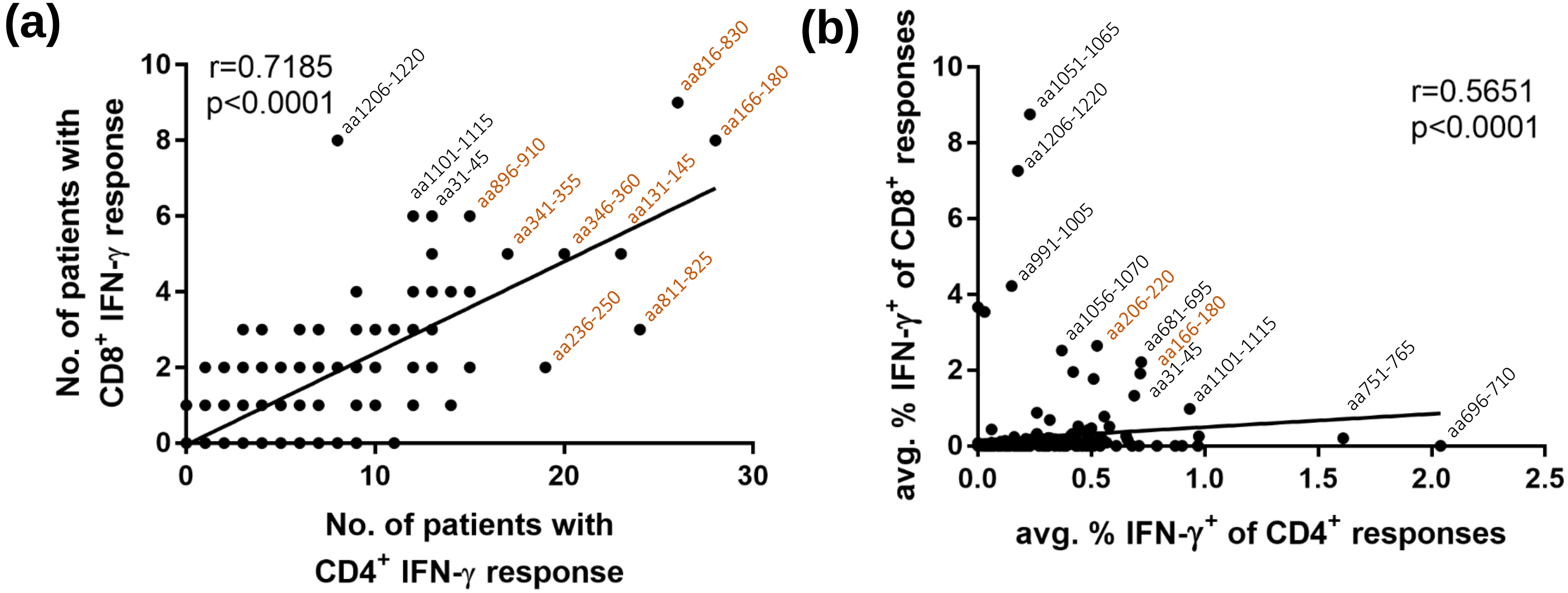

Supplementary Figure 9. Correlation analysis of number and magnitude of CD4⁺ and CD8⁺ T-cell responses towards each peptide.
The number **(a)** and average magnitude **(b)** of the CD4⁺ and CD8⁺ T-cell responses for each peptide correlate significantly. Each dot represents one of the 15-mer peptides. For peptides frequently recognized **(a)** or recognized with high average magnitudes **(b)** by CD4⁺ and CD8⁺ T-cells, the amino acids are given. Orange color indicates peptides included in the pool of most frequently detected peptides. For reference see Figure 3b.

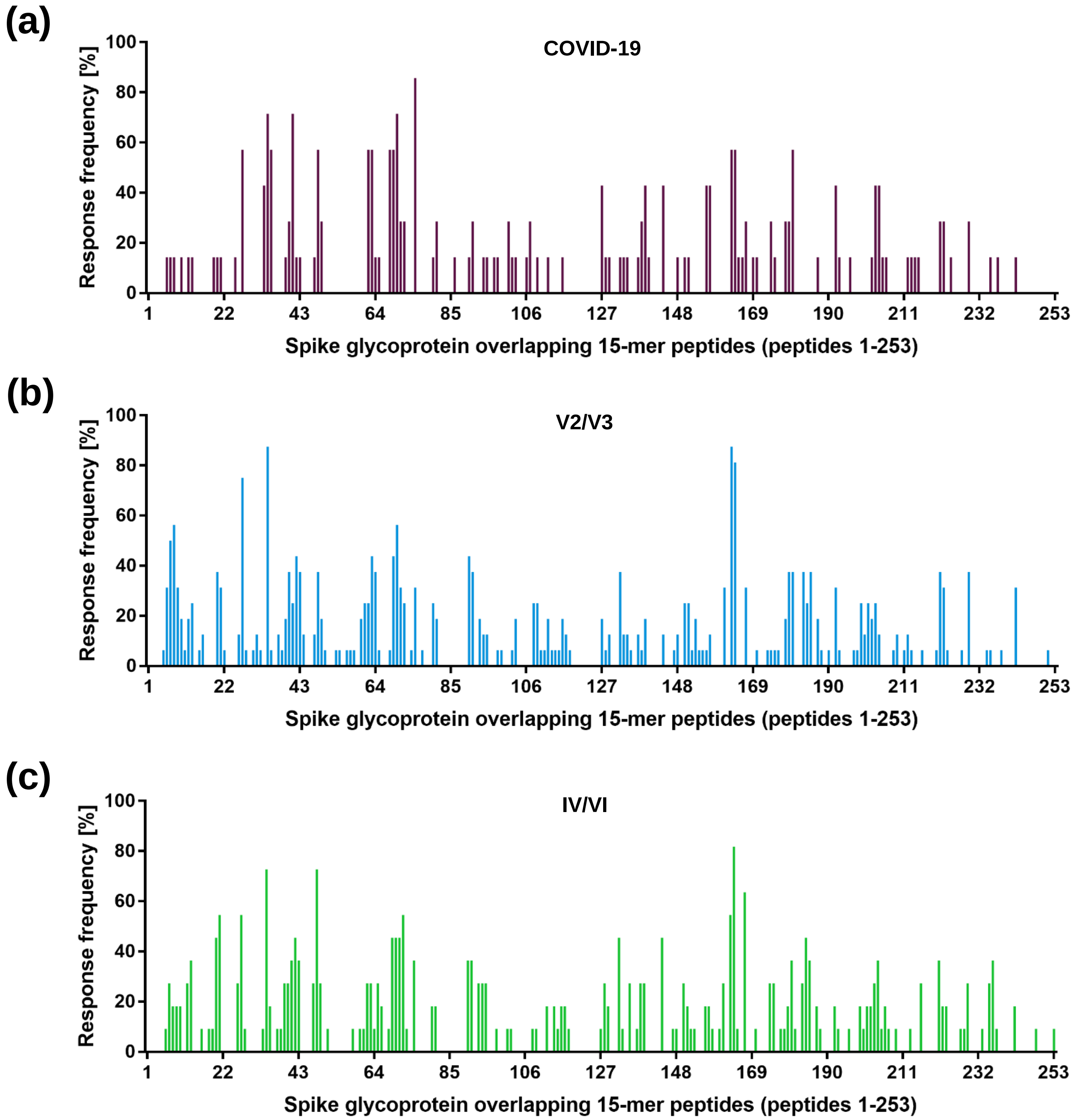

Supplementary Figure 10. Response frequency to the individual spike 15-mer peptides subdivided by study groups. CD4^{+} T-cell response frequencies of COVID-19 patients **(a)**, vaccinated individuals **(b)**, and individuals with both vaccination and infection **(c)** to every of the 253 overlapping 15-mer peptides covering the spike glycoprotein.

peptide#	aa position	Sequence															RF
**Most frequently detected peptides of the spike glycoprotein - COVID-19**																	
27	131-145	C	E	F	Q	F	C	N	D	P	F	L	G D	V Del	Y Del	Y Del	62.50%
34	166-180	C	T	F	E	Y	V	S	Q	P	F	L	M	D	L	E	75.00%
35	171-185	V	S	Q	P	F	L	M	D	L	E	G	K	Q	G	N	50.00%
41	201-215	F	K	I	Y	S	K	H	T	P	I V	N Del	L I	V G	R insEPE	D	62.50%
48	236-250	T	R	F	Q	T	L	L	A	L	H	R	S	Y	L	T	62.50%
62	306-320	F	T	V	E	K	G	I	Y	Q	T	S	N	F	R	V	50.00%
63	311-325	G	I	Y	Q	T	S	N	F	R	V	Q	P	T	E	S	50.00%
68	336-350	C	P	F	G D/H	E	V	F	N	A	T	R	F	A	S	V	50.00%
69	341-355	V	F	N	A	T	R	F	A	S	V	Y	A	W	N	R	62.50%
70	346-360	R	F	A	S	V	Y	A	W	N	R	K	R	I	S	N	75.00%
75	371-385	S L/F	A	S P	F	S F	T A	F	K	C	Y	G	V	S	P	T	75.00%
163	811-825	K	P	S	K	R	S	F	I	E	D	L	L	F	N	K	50.00%
164	816-830	S	F	I	E	D	L	L	F	N	K	V	T	L	A	D	50.00%
180	896-910	I	P	F	A	M	Q	M	A	Y	R	F	N	G	I	G	62.50%

peptide#	aa position	Sequence															RF
**Most frequently detected peptides of the spike glycoprotein - V2/V3**																	
7	31-45	S	F	T	R	G	V	Y	Y	P	D	K	V	F	R	S	50.00%
8	36-50	V	Y	Y	P	D	K	V	F	R	S	S	V	L	H	S	56.25%
27	131-145	C	E	F	Q	F	C	N	D	P	F	L	G D	V Del	Y Del	Y Del	75.00%
34	166-180	C	T	F	E	Y	V	S	Q	P	F	L	M	D	L	E	87.50%
42	206-220	K	H	T	P	I V	N Del	L I	V G	R insEPE	D	L	P	Q	G	F	43.75%
63	311-325	G	I	Y	Q	T	S	N	F	R	V	Q	P	T	E	S	43.75%
69	341-355	V	F	N	A	T	R	F	A	S	V	Y	A	W	N	R	43.75%
70	346-360	R	F	A	S	V	Y	A	W	N	R	K	R	I	S	N	56.25%
90	446-460	G S	G	N	Y	N	Y	L Q/R	Y	R	L	F	R	K	S	N / K	43.75%
163	811-825	K	P	S	K	R	S	F	I	E	D	L	L	F	N	K	87.50%
164	816-830	S	F	I	E	D	L	L	F	N	K	V	T	L	A	D	81.25%

peptide#	aa position	Sequence															RF
**Most frequently detected peptides of the spike glycoprotein - IV/VI**																	
20	96-110	E	K	S	N	I	I	R	G	W	I	F	G	T	T	L	45.45%
21	101-115	I	R	G	W	I	F	G	T	T	L	D	S	K	T	Q	54.55%
27	131-145	C	E	F	Q	F	C	N	D	P	F	L	G D	V Del	Y Del	Y Del	54.55%
34	166-180	C	T	F	E	Y	V	S	Q	P	F	L	M	D	L	E	72.72%
42	206-220	K	H	T	P	I V	N Del	L I	V G	R insEPE	D	L	P	Q	G	F	45.45%
48	236-250	T	R	F	Q	T	L	L	A	L	H	R	S	Y	L	T	72.72%
69	341-355	V	F	N	A	T	R	F	A	S	V	Y	A	W	N	R	45.45%
70	346-360	R	F	A	S	V	Y	A	W	N	R	K	R	I	S	N	45.45%
71	351-365	Y	A	W	N	R	K	R	I	S	N	C	V	A	D	Y	45.45%
72	356-370	K	R	I	S	N	C	V	A	D	Y	S	V	L	Y	N	54.55%
132	656-670	V	N	N	S	Y	E	C	D	I	P	I	G	A	G	I	45.45%
144	716-730	T	N	F	T	I	S	V	T	T	E	I	L	P	V	S	45.45%
163	811-825	K	P	S	K	R	S	F	I	E	D	L	L	F	N	K	54.55%
164	816-830	S	F	I	E	D	L	L	F	N	K	V	T	L	A	D	81.82%
167	831-845	A	G	F	I	K	Q	Y	G	D	C	L	G	D	I	A	63.64%
184	916-930	L	Y	E	N	Q	K	L	I	A	N	Q	F	N	S	A	45.45%

Supplementary Figure 11. Identification of the most frequently detected peptides by CD4$^+$ T cells subdivided by study groups. Peptide numbers, corresponding amino acid positions and sequences as well as response frequencies of the most frequently detected peptides. Mutations in the variants of concern (VoC) are highlighted: Mutations found only in BA.1 are in blue, mutations found in BA.1, BA.2, BA.2.12, BA.4/5 and BA.2.75 are in red, mutations found in BA.2, BA.2.12, BA.4/5 and BA.2.75 are in green. Mutations found in BA.1, BA.2, BA.2.12 and BA4./5 are in light blue. Mutations only found in BA.2.12 are in orange and mutations only found in BA.4/5 are in violet. Mutations found in BA.1 and BA.2.75 are in grey, mutations only found in BA.2.75 are in pink.

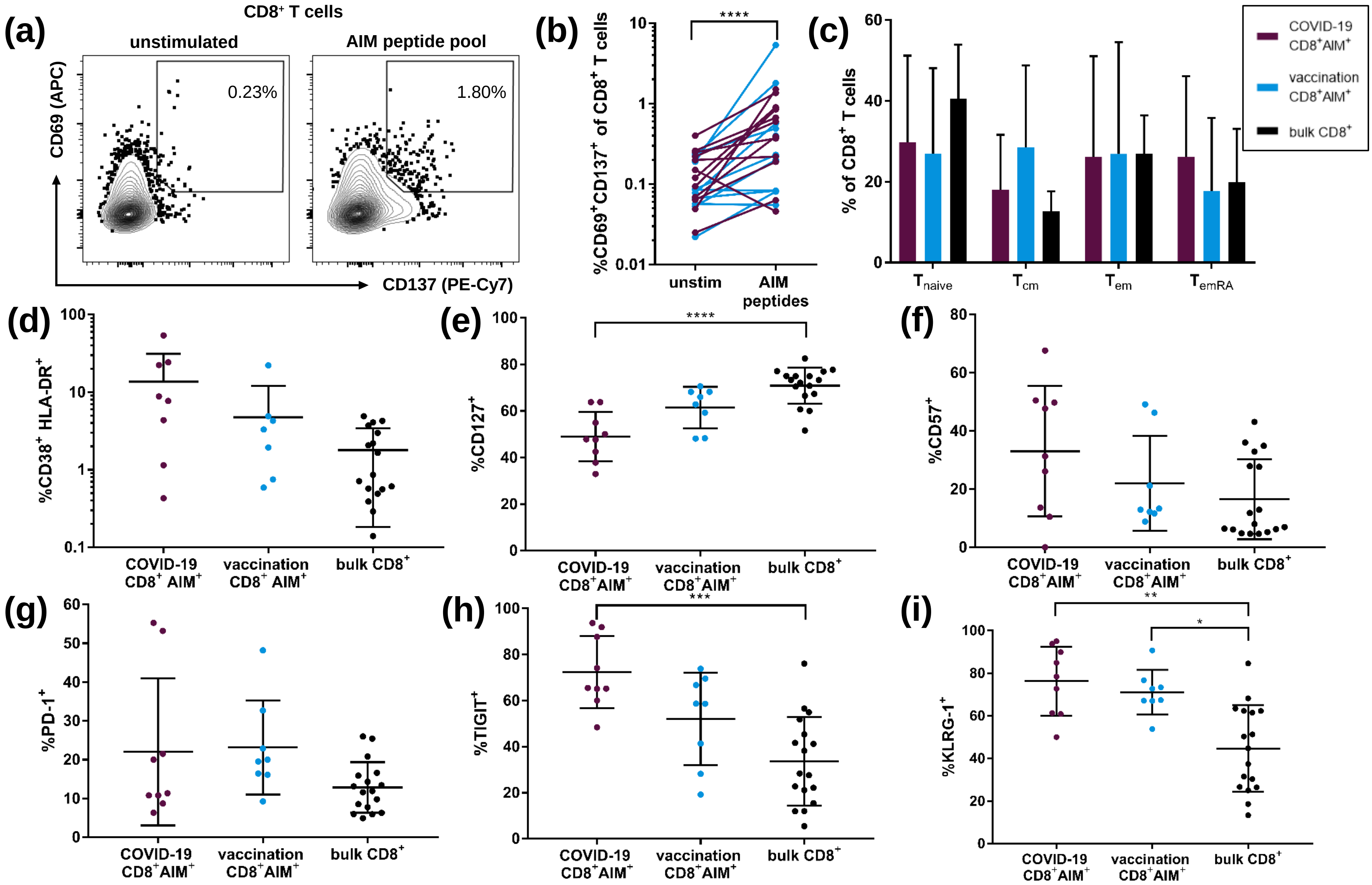

Supplementary Figure 12. Frequencies and phenotype of AIM⁺ CD8⁺ T cells in response to a spike glycoprotein peptide pool. Thawed PBMC were stimulated for 18h with a peptide pool or SEB (positive control) or were left untreated (negative control) and analyzed by flow cytometry. Antigen-reactive CD8⁺ T cells were defined as CD69⁺CD137⁺ (**a**). After stimulation with the peptide pool, an increase in AIM⁺ (CD69⁺CD137⁺) CD8⁺ T cells could be observed in most individuals except for a few non-responders (**b**). Non-responders were excluded from further analyses. Memory phenotype of AIM⁺ CD8⁺ T cells of individuals with COVID-19 or vaccination in comparison to bulk CD8⁺ T cells (**c**). AIM⁺ CD8⁺ T cells of individuals with COVID-19 and vaccinated individuals were compared to bulk CD8⁺ T cells with regard to their expression of activation markers CD38 and HLA-DR (**d**), CD127 (**e**), CD57 (**f**), PD-1 (**g**), TIGIT (**h**) and KLRG-1 (**i**).

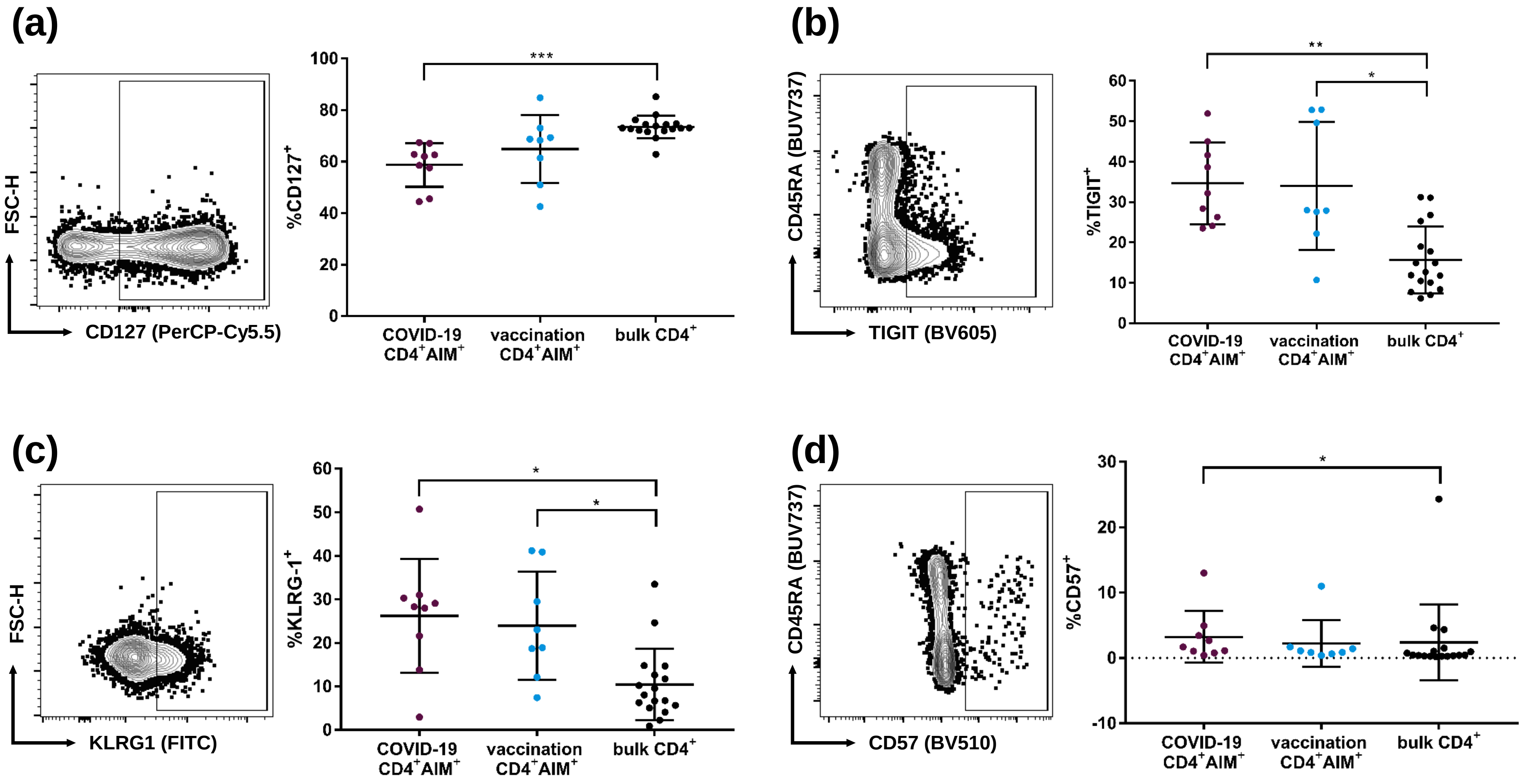

Supplementary Figure 13. Phenotype of AIM+ CD4+ T cells in response to a spike glycoprotein peptide pool. AIM+ CD4+ T cells of individuals with COVID-19 show significantly reduced expression of CD127 **(a)** and significantly higher proportions of TIGIT+ **(b)** and KLRG1+ **(c)** frequencies compared to bulk CD4+ T cells. CD57 expression is not significantly different between AIM+ and bulk CD4+ T cells **(d)**.

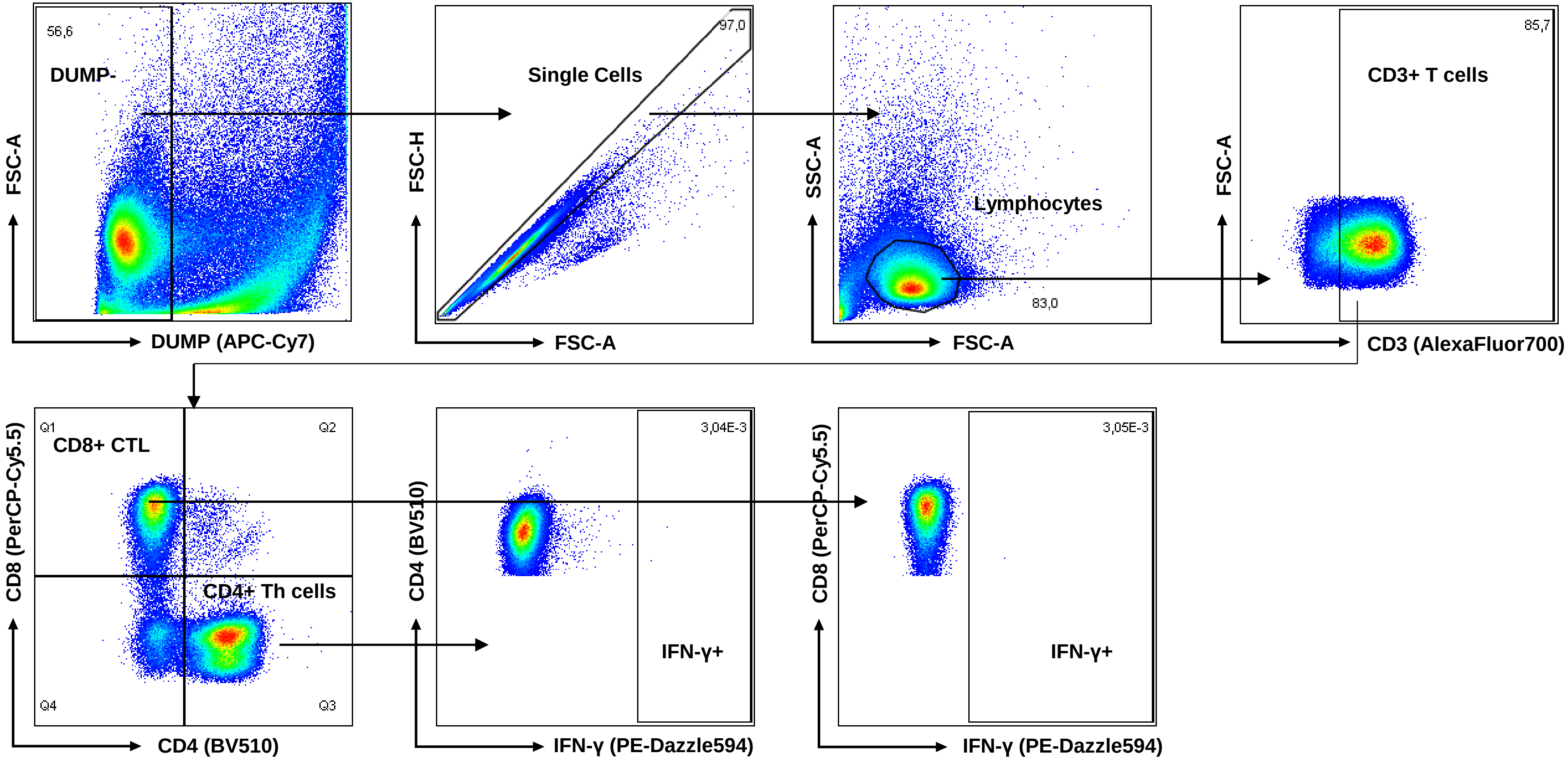

Supplementary Figure 14. Gating strategy for identification of IFN-γ producing $CD4^+$ and $CD8^+$ T cells. First, dead cells, $CD14^+$ and $CD19^+$ cells (DUMP) as well as doublets were excluded. $CD4^+$ T helper cells were identified as $CD3^+$ $CD4^+$ $CD8^-$ Lymphocytes and $CD8^+$ cytotoxic T cells were identified as $CD3^+$ $CD4^-$ $CD8^+$ Lymphocytes. Both Subsets were analyzed for IFN-γ expression.
